# Plant Immune System Activation Upon Citrus Leprosis Virus C Infection Is Mimicked by the Ectopic Expression of the P61 Viral Protein

**DOI:** 10.3389/fpls.2020.01188

**Published:** 2020-08-07

**Authors:** Gabriella D. Arena, Pedro Luis Ramos-González, Bryce W. Falk, Clare L. Casteel, Juliana Freitas-Astúa, Marcos A. Machado

**Affiliations:** ^1^ Laboratório de Biotecnologia, Centro de Citricultura Sylvio Moreira, Instituto Agronômico de Campinas, Cordeirópolis, Brazil; ^2^ Escola Superior de Agricultura Luiz de Queiroz (ESALQ), Universidade de São Paulo, Piracicaba, Brazil; ^3^ Laboratório de Biologia Molecular Aplicada, Instituto Biológico, São Paulo, Brazil; ^4^ Department of Plant Pathology, University of California, Davis, Davis, CA, United States; ^5^ School of Integrative Plant Science, Cornell University, Ithaca, NY, United States; ^6^ Laboratório de Virologia Vegetal, Embrapa Mandioca e Fruticultura, Cruz das Almas, Brazil

**Keywords:** *Cilevirus*, RNA-Seq, plant–virus interaction, hypersensitive response, salicylic acid, jasmonic acid, *Arabidopsis thaliana*, *Nicotiana benthamiana*

## Abstract

Citrus leprosis virus C (CiLV-C, genus *Cilevirus*, family *Kitaviridae*) is an atypical virus that does not spread systemically in its plant hosts. Upon its inoculation by *Brevipalpus* mites, only localized lesions occur, and the infection remains limited to cells around mite feeding sites. Here, we aimed to gain insights into the putative causes of viral unfitness in plants by expanding the limited knowledge of the molecular mechanisms underlying plant/kitavirid interactions. Firstly, we quantified the CiLV-C viral RNAs during the infection in *Arabidopsis thaliana* plants using RT-qPCR and systematized it by defining three stages of distinguishing subgenomic and genomic RNA accumulation: i) 0–24 h after infestation, ii) 2–4 days after infestation (dai), and iii) 6–10 dai. Accordingly, the global plant response to CiLV-C infection was assessed by RNA-Seq at each period. Results indicated a progressive reprogramming of the plant transcriptome in parallel to the increasing viral loads. Gene ontology enrichment analysis revealed the induction of cell growth-related processes at the early stages of the infection and the triggering of the SA-mediated pathway, ROS burst and hypersensitive response (HR) at the presymptomatic stage. Conversely, infected plants downregulated JA/ET-mediated pathways and processes involved in the primary metabolism including photosynthesis. Marker genes of unfolded protein response were also induced, suggesting a contribution of the endoplasmic reticulum stress to the cell death caused by the viral infection. Finally, we transiently expressed CiLV-C proteins in *Nicotiana benthamiana* plants to undertake their roles in the elicited plant responses. Expression of the CiLV-C P61 protein consistently triggered ROS burst, upregulated SA- and HR-related genes, increased SA levels, reduced JA levels, and caused cell death. Mimicry of responses typically observed during CiLV-C–plant interaction indicates P61 as a putative viral effector causing the HR-like symptoms associated with the infection. Our data strengthen the hypothesis that symptoms of CiLV-C infection might be the outcome of a hypersensitive-like response during an incompatible interaction. Consequently, the locally restricted infection of CiLV-C, commonly observed across infections by kitavirids, supports the thesis that these viruses, likely arising from an ancestral arthropod-infecting virus, are unable to fully circumvent plant defenses.

## Introduction

Viruses that accomplish plant systemic infections replicate in the entry cell and use it as the source for local infections in contiguous cells, invade, and spread throughout the plant *via* the vascular system. Upon challenging a resistant host, viral multiplication and/or movement may be compromised. Differently from the majority of plant viruses, citrus leprosis virus C (CiLV-C, genus *Cilevirus*, family *Kitaviridae*) is unable to systemically infect any of its natural or experimental host species, even those belonging to distant plant families (Nunes et al., 2012; Arena et al., 2013; Garita et al., 2014). Invariably, CiLV-C remains restricted to cells around the vector-mediated inoculation sites, where symptoms of viral infection are chlorotic or necrotic spots (Bastianel et al., 2010). Despite the constraint in systemic infection for CiLV-C, it causes citrus leprosis, the most important viral disease affecting the citrus industry in Brazil, the world leader in sweet orange production. Annually, prevention and control of citrus leprosis cost approximately 50 million dollars, mainly for the chemical control of the viral vector, mites of the species *Brevipalpus yothersi* (Bassanezi et al., 2019). Endemic in the Americas, CiLV-C has spread throughout the main citrus-producing areas of the Latin American subregion (Ramos-González et al., 2018).

CiLV-C has two positive (+) sense single-stranded genomic RNA molecules with six open reading frames (ORFs). RNA1 (8,745 nts) harbors two ORFs encoding the RNA-dependent RNA polymerase (RdRp) and the putative coat protein (P29). RNA2 (4,986 nts) presents four ORFs encoding the putative movement protein (MP) and the P15, P61, and P24 proteins with unknown functions (Locali-Fabris et al., 2006; Pascon et al., 2006). While the *p15* ORF is considered an orphan gene with no homologs in any other viral species, *p61* and *p24* are taxonomically restricted ORFs also present in insect-infecting negeviruses and other nege-like viruses (Tautz and Domazet-Lošo, 2011; Kuchibhatla et al., 2014). P61 and P24 from kitaviruses and their related insect-infecting viruses show conserved structural features such as transmembrane domains and signal peptides (Kuchibhatla et al., 2014). CiLV-C RNA1 drives the transcription of one subgenomic RNA (sgRNA) of 0.7 kb for the expression of the *p29* gene, and RNA2 generates three coterminal sgRNAs of 3, 1.5, and 0.6 kb from where P61, MP, and P24 are translated, respectively (Pascon et al., 2006).

The reasons behind CiLV-C’s inability to systemically infect plants are still unknown. There is speculation that the viral unfitness might be due to defective viral movement factors, an effective plant immune system that CiLV-C is unable to overcome, or even a combination of these and other ignored factors. Since a recent study revealed the functionality of cilevirus MP (Leastro et al., submitted), alternative hypotheses, such as those involving efficacious plant defenses preventing CiLV-C spread, gain strength. Plant innate defense mechanisms against pathogens are based on a two-layered immune system that uses cell surface receptors and intracellular plant resistance (R) proteins to respectively recognize pathogen-associated molecular patterns (PAMPs) or effectors (Cui et al., 2015; Couto and Zipfel, 2016; Garcia-Ruiz, 2019). Metabolic changes induced during plant defense can result in a burst of reactive oxygen species (ROS) that may culminate in a hypersensitive response (HR) (Xia et al., 2015). The transcriptional reprogramming resulting in the defense responses is mediated by the action of interconnected phytohormonal-dependent pathways and directed according to the nature of the injury. Typically, the salicylic acid (SA) pathway confers resistance to biotrophic pathogens and antagonizes the jasmonate/ethylene (JA/ET) pathways that in turn induce defenses against herbivores and necrotrophic pathogens (Pieterse et al., 2012). The SA pathway is involved in the activation of the HR, acting together with ROS molecules to trigger the resistance response (Xia et al., 2015). Furthermore, activation of the plant immune system and the induction of HR have been linked to endoplasmic reticulum (ER) stress (Poór et al., 2019). The excessive accumulation of proteins in the ER triggers the unfolded protein response (UPR), a mechanism that prevents the dangerous accumulation of unfolded proteins, but surpassed a threshold, UPR leads to a chronic stress condition that eventually triggers HR (Williams et al., 2014).

Several viral proteins have been identified as elicitors of the plant immune system. For instance, the P0 protein from poleroviruses elicits an HR that is associated with the *Nicotiana glutinosa* protein RPO1 (Resistance to Poleroviruses 1), a likely immune receptor of P0 (Wang et al., 2015). Likewise, the P38 protein from turnip crinkle virus (TCV) is recognized by the *Arabidopsis thaliana* R protein HRT (HR to TCV), which activates an HR-mediated resistance (Cooley et al., 2000; Pumplin and Voinnet, 2013). In a clear correlation between over-accumulation of viral proteins in the ER and HR, the transient expression of the P25 protein from potato virus X in *N. benthamiana* plants induces ER stress and UPR, leading to ER collapse and cell death (Aguilar et al., 2019). Besides triggering HR, viral proteins can modulate hormonal defense pathways to establish mutualism with its vector (Casteel and Falk, 2016). For example, the NIa-Pro (Nuclear inclusion a-protease) protein from turnip mosaic virus (TuMV) interferes with ET-mediated responses, resulting in defense suppression and, consequently, the enhanced performance of its vector, the aphid *Myzus persicae* (Casteel et al., 2015). Viruses that depend on vectors to move from infected to healthy host plants use this strategy of decrease antiherbivory defense as an effective means to improve their transmissibility (Abe et al., 2012).

A previous study conducted in *A. thaliana* plants revealed that CiLV-C infection triggers ROS burst and cell death, induces the classical antiviral mechanisms of RNA silencing and SA pathway, suppresses the JA-dependent response, and favors the colonization of the mite vector. A preliminary model of the interaction using this information was depicted (Arena et al., 2016), but many underlying mechanisms of CiLV-C infection remain to be uncovered, for instance, the kinetics of viral accumulation in infected plants, the global plant response to the virus infection, and the viral effector that triggers such response. Here we aim to expand our knowledge on the molecular plant/CiLV-C interplay. Firstly, to describe the CiLV-C accumulation along the course of the infection, we quantify viral genomic and subgenomic RNAs using RT-qPCR. Then, to unravel novel mechanisms of plant response to the viral infection, we evaluate the transcriptome profile of infected *A. thaliana* plants by RNA-Seq. Finally, to test the hypothesis that specific CiLV-C proteins could trigger plant responses to the viral infection, the putative elicitor activity of each virus protein was assessed by expressing them in *Nicotiana benthamiana* plants. The current work contributes to the identification of mechanisms involved in the development of citrus leprosis disease and provides insights around the atypical restraint of the systemic movement of CiLV-C. In practical terms, we provide data supporting a comprehensive plant transcriptome analysis that can be further explored to unravel common or unique mechanisms of plant gene expression operated during the plant infection by kitavirids.

## Materials and Methods

### Plant Material

Seeds from *A. thaliana* ecotype Columbia (Col-0) were obtained from the Arabidopsis Biological Resource Center (ABRC, http://www.arabidopsis.org). *A. thaliana* and *N. benthamiana* plants were grown in a controlled growth chamber Adaptis AR A1000 (Conviron, Winnipeg, Canada) at 23 ± 2°C and a 12 h photoperiod. Four-week-old plants were used in the experiments.

### Mite Rearing

The population of mites was initially obtained from a single female collected from a citrus orchard and identified as *B. yothersi* using phase-contrast microscopy as reported elsewhere (Beard et al., 2015). Nonviruliferous mites were reared onto the unripe fruits of Persian lime (*Citrus latifolia* Tanaka), a genotype immune to CiLV-C. Viruliferous mites were obtained by transferring the nonviruliferous mites from the Persian lime to sweet orange fruits with citrus leprosis symptoms infected with CiLV-C strain SJP (Ramos-González et al., 2016). Fruits were prepared as described before (Rodrigues et al., 2007). Mites were reared for several generations and were evaluated for the presence of CiLV-C by RT-PCR (Locali et al., 2003) before their use in the experiments.

### Kinetics of CiLV-C Accumulation Experiment

Quantification of CiLV-C RNA loads was performed in *A. thaliana* plants infested with *B. yothersi* viruliferous mites at ¼, ½, 1, 2, 4, 6, 8, and 10 dai. *A. thaliana* plants were infested with 15 mites (five per each of three rosette leaves), transferred with a brush under a stereoscopic microscope. Each time point had 10 biological replicates. Harvested leaves were flash-frozen in liquid N_2_ and stored at −80°C until RNA extraction. Plant RNA was purified with the RNeasy Plant Mini Kit (Qiagen, Venlo, Netherlands); RNA concentration and purity (A_260_/A_280_) were determined in NanoDrop ND-8000 micro-spectrophotometer (Thermo Scientific, Waltham, USA), and cDNA was synthesized using RevertAid H Minus First Strand cDNA Synthesis Kit (Thermo Scientific, Waltham, MA, USA).

### Absolute Quantification of CiLV-C *p29* and *RdRp* Genes

Absolute quantification of *p29* and *RdRp* was assessed by RT-qPCR using TaqMan assays. Reaction mixes were prepared with the TaqMan^®^ Fast Universal PCR MasterMix 2× kit, as recommended by the manufacturer (Thermo Scientific, Waltham, MA, USA). Amplifications were carried out in a 7500 Fast Real-Time PCR System device (Thermo Scientific, Waltham, MA, USA). Samples were analyzed in triplicates and no-template controls were included to check for contaminations. *Cycle quantification* (Cq) values from infected samples were compared with the standard curves to determine absolute quantities of CiLV-C *p29* and *RdRp* molecules. Quantities of each gene at different time points were statistically compared using one-way ANOVA and Student’s t-test (*α* ≤ 0.05).

### Relative Quantification of CiLV-C Genes

Relative quantification of all CiLV-C ORFs (*p29*, *RdRp*, *p15*, *p61*, *p24*, and *MP*), the putative *p7* ORF, and the intergenic region (IR) was assessed by RT-qPCR using the GoTaq dsDNA binding dye. qPCR assays were prepared with 3 ng of cDNA, 6.5 ul of GoTaq qPCR Master Mix (Promega, Madison, WI, USA), and 120 nM of each gene-specific primer pair. Each cDNA sample was analyzed in duplicates, and melting curves were included. Primer pair efficiency (E) and Cq value were determined for each reaction using Real-time PCR Miner (Zhao and Fernald, 2005). The Cq value of each sample, expressed as the mean of the two technical replicates, was converted into relative quantities (RQs) using the function RQ = E^ΔCq^, where ΔCq is the difference between the lowest Cq value across all samples for the evaluated gene and the Cq value of a given sample. Normalized-relative quantity (NRQ) of each sample was calculated as the ratio of the sample RQ and the reference gene (*A. thaliana* *SAND family protein* gene) RQ. Individual fold change values were determined by dividing the sample NRQ by the mean NRQ of samples of the calibrator, that is, plants collected at the time point with the lowest amount of the specific molecule. Quantities of each molecule at different time points were statistically compared using one-way ANOVA and Student’s t-test (*α* ≤ 0.05).

### RNA-Seq Time-Course Experiment

*Arabidopsis thaliana* plants were infested with 15 nonviruliferous or viruliferous mites (five mites per each of three rosette leaves). Infested leaves were collected at 6 h after infestation (hai) and 2 and 6 days after the infestation (dai). Sixteen plants were infested per treatment per time point, and leaves from two plants were pooled, totaling eight biological replicates. Another set of plants was kept with viruliferous mites for eight days, when symptoms were visible, to confirm virus infection. Harvested leaves were flash-frozen in liquid N_2_ and stored at −80°C until RNA extraction. Plant RNA was purified with the RNeasy Plant Mini Kit (Qiagen, Venlo, Netherlands) and treated with RNAse-free DNAse (Qiagen, Venlo, Netherlands) to remoe plant DNA. RNA purity (A_260_/A_280_ ~ 2.0) and integrity (RIN > 8) were confirmed in NanoDrop ND-8000 micro-spectrophotometer (Thermo Scientific, Waltham, USA) and Bioanalyzer 2100 (Agilent Technologies, Santa Clara, USA), respectively. CiLV-C presence in plants infested with viruliferous mites or its absence in those infested with nonviruliferous mites was confirmed by RT-PCR (Locali et al., 2003). RNA extracts from two samples (100 ng/ul each) were pooled, totaling four replicates per treatment (CiLV-C and mock) per time point for the RNA-Seq. cDNA libraries were prepared with Illumina TruSeq Stranded mRNA Library Prep Kit (Illumina, San Diego, USA). Sequencing was performed in an Illumina HiSeq 2500 system (Illumina, San Diego, USA) using HiSeq SBS v4 High Output Kit (Illumina, San Diego, USA). Paired-end reads of 2 × 125 bp were generated.

### Bioinformatics Analysis of RNA-Seq Data

RNA-Seq data were analyzed following the pipeline from Anders et al. (2013) with some modifications previously described (Arena et al., 2018). The biological variability of the samples was assessed by principal component analysis (PCA) and hierarchical clustering (using the *Euclidean* distance metric and *Ward’s* clustering method). Differentially expressed genes (DEGs) between CiLV-C and mock treatments were identified at each time point using the package DESeq2 (Love et al., 2014). *False Discovery Rate* (FDR) correction for multiple comparisons was applied. DEGs with corrected *p*-values ≤ 0.05 and |log2FC| ≥ 0.5 were classified as differentially expressed. GO Enrichment Analysis was performed on DEGs to elucidate mechanisms potentially involved in the CiLV-C infection and symptoms development. A gene set was defined as all DEGs identified at each set (2 dai/upregulated, 2 dai/downregulated, 6 dai/upregulated, and 6 dai/downregulated), and the universe comprised all genes of the *A. thaliana* TAIR10 genome. Overrepresented Biological Processes (BPs) were identified by a hypergeometric test (FDR-adjusted *p*-values ≤ 0.001). GO networks were generated in Cytoscape using the app BinGO (Maere et al., 2005).

### Identification of Enriched Transcription Factors

Enriched TFs were assessed on up and downregulated DEGs from 2 and 6 dai using two approaches. First, genes coding for TFs within DEGs were identified by searching on the PlantTFDB database (Jin et al., 2017) and overrepresented TF families on each set of genes were assessed using a hypergeometric test (*α* ≤ 0.01). Second, individual TFs with targets enriched within DEGs were identified using the TF enrichment tool (Jin et al., 2017), based on both the presence of *cis*-regulatory elements in the sequences of the DEGs assessed and literature mining. TFs with enriched targets were further grouped according to their families.

### Validation of Gene Expression Data by RT-qPCR

A new time-course experiment was set with *A. thaliana* Col-0 plants infested with viruliferous and nonviruliferous mites. Leaf samples were collected at 6 hai, 2 dai, and 6 dai. For each time point, plants were grouped in sets of 16 individuals assigned to each treatment (CiLV-C and mock). Plants were infested with 15 mites (five mites per each of three rosette leaves). Infested leaves were collected at each time point, and leaves from two plants were pooled, totaling eight biological replicates per treatment per time point. Leaf collection, RNA extraction, and quantification were carried out as previously indicated in this section. RNA quality was confirmed in 1.2% agarose gels. cDNA was generated using RevertAid H Minus First Strand cDNA Synthesis Kit as described by the manufacturer (Thermo Scientific, Waltham, MA, USA). qPCR assays were prepared with 3 ng of cDNA, 6.5 ul of GoTaq qPCR Master Mix (Promega, Madison, WI, USA), and 120 nM of each gene-specific primer pair (**Supplementary Table 8**). Each cDNA sample was analyzed in triplicate, and melting curves were included. Primer pairs’ efficiency and Cq value were determined for each reaction using Real-time PCR Miner (Zhao and Fernald, 2005). Gene expression analyses were performed using the ΔCq model with efficiency correction and multiple reference genes (Hellemans et al., 2007) as previously described (Arena et al., 2016). The difference between infected and mock samples within each time point was assessed using Student’s t-test (*α* ≤ 0.05).

### Cloning of CiLV-C ORFs in Expression Vectors

CiLV-C ORFs were amplified from pregenerated clones using a HiFi polymerase (Thermo Scientific, Waltham, USA), and specific primers were designed to add restriction sites to the ends of each amplicon (**Supplementary Table 9**). The amplicons were digested and cloned in an intermediary vector based on the backbone of the pUC19 cloning vector (New England Biolabs, Ipswich, USA). Each transcriptional unit comprised the 35s cauliflower mosaic virus promoter driving constitutive expression of the viral ORF, the Ω fragment from TMV as a translational enhancer, the convenient CiLV-C ORF, and the *nopaline synthase* terminator. After assembly, each transcription unit was transferred from the intermediary vector to a pCambia 2300 binary vector (Marker Gene Technologies, Ipswich, USA). Final constructions were digested with the endonucleases *Xba*I and *Xho*I for validation of their identity. After the identification of the P61 elicitor activity (methods described below), new expression clones were assembled to express 3xFLAG C-terminal tagged P61 protein under the control of a dexamethasone (DEX)-inducible promoter. Somewhat similar cloning procedures were performed using the Gateway system (Thermo Scientific, Waltham, USA) to construct GFP-expressing clones as negative controls. Specific primers (**Supplementary Table 9**) were designed to include four Gs and the 25 bp of the attB regions in the 5′ end (for efficient Gateway cloning), the stop codon was removed (for fusion with the 3xFLAG), and nucleotides were added (to maintain the proper reading frame with the FLAG tag). Genes were amplified using the *Phusion High-Fidelity DNA Polymerase* (New England Biolabs Ipswich, USA). The amplicons were purified and cloned in the donor vector pDONR207 (Thermo Scientific, Waltham, USA). Entry clones were purified and confirmed by nucleotide sequencing. Transcriptional units were transferred by recombination to a Gateway-compatible version of the pTA7001 destination vector (Aoyama et al., 1997; Li et al., 2013), with a C-terminal 3xFLAG (DYKDHDGDYKDHDIDYKDDDDK) and a DEX-inducible promoter. Expression clones were purified and sequenced to confirm their identity. To generate the *p61* construct containing the frameshift mutant (p61Fs), the *p61* gene was amplified from the previously constructed vector using a specific forward primer (**Supplementary Table 9**) designed to introduce two extra nucleotides following the start codon. The amplicons were cloned in the pDONR207, and the transcriptional units were further transferred to the Gateway-compatible version of the pTA7001 destination vector. Expression clones were purified and confirmed by sequencing.

### Transient Expression Assays in *Nicotiana benthamiana* Leaves

Plasmids containing the studied ORF were inserted into the *Agrobacterium tumefaciens* strain GV3101. Recombinant *A. tumefaciens* was cultivated overnight in 5 ml of Luria-Bertani (LB) medium containing kanamycin (30 µg/ml) and rifampicin (50 µg/ml). Fresh media (Kn, Rif, and 150 μM acetosyringone) were inoculated with 1/10 (v/v) of the pre-inoculum until reaching a 0.8–1 OD_600_ nm. Cells were harvested by centrifugation and further incubated in the infiltration buffer (10 mM MES, pH 5.6, 10 mM MgCl_2_, 150 μM acetosyringone) in the dark for 4 h. Infiltrations were carried out using a syringe in leaves of four- to six-leaf stage plants. In parallel, *N. benthamiana* plants were infiltrated with the empty vectors, GFP-expressing clones (negative controls), and the infiltration buffer (blank). When pTA7001 expression clones were used, infiltrated leaves were sprayed with 30 uM DEX and collected at 1, 2, and 3 days after induction. Cells were lysed in the presence of NP40-based buffer (150 mM NaCl, 1% NP40, 50 mM Tris-HCl), diluted in a protein disruption buffer (136 mM DTT, 192 mM Tris, 45 mg/ml SDS, 50 ug/ml bromophenol blue, 10 M urea), heated at 95°C, and loaded in SDS-PAGE gels. The fusion proteins were assessed by Western Blot probed with anti-DYKDDDDK-HRP conjugate (Miltenyi Biotec, Auburn, USA) at a concentration of 1:2,000 (**Figure 7F**). Leaves infiltrated with the construction for the expression of P61, empty vector, or the expression of GFP were collected for histochemical detection of H_2_O_2_, expression analysis of marker genes, and quantification of defense hormones. H_2_O_2_ was visualized by leaf staining with diaminobenzidine (DAB) as reported elsewhere (Ilarduya et al., 2003). The expression profiles of SA- and HR-related genes were assessed by RT-qPCR as described in the topic *Validation of Gene Expression Data by RT-qPCR*, with primer pairs described by Li et al. (2012). The SA and JA contents were quantified by LC-MS/MS as previously described (Arena et al., 2018). *N. benthamiana* plants were inoculated with CiLV-C, and symptoms were compared with those from plants agroinfiltrated with the plasmids containing the viral ORFs. Lesions on CiLV-C-infected sweet orange fruits were collected, ground in a mortar, and the sap was mechanically inoculated into carborundum-dusted leaves of four-week-old *N. benthamiana* plants.

## Results

### Amounts of CiLV-C-Specific RNAs Continuously Increase Through the First Ten Days of Leaf Infection, a Period During Which Three Distinct Viral Accumulation Stages Are Distinguished

The kinetics of accumulation of CiLV-C-specific RNAs in *A. thaliana* infected leaves was evaluated by RT-qPCR. At first, the targets for TaqMan-based assays were two regions of the CiLV-C RNA1, one within the gene *p29*, which is present in the genomic and sgRNA and codes for the putative capsid protein, and another inside the ORF *RdRp* that is directly translated from the genomic RNA and codes for the viral replicase.

**Figure 1:**
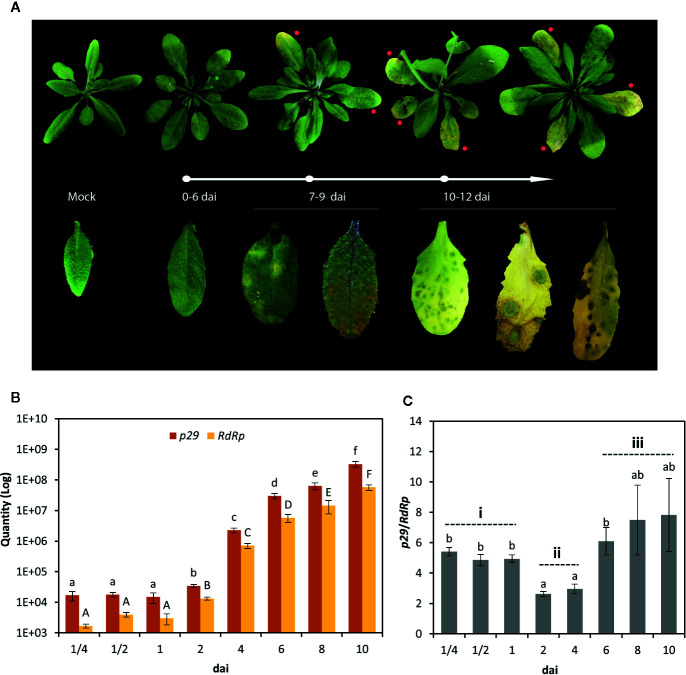
CiLV-C infection in *Arabidopsis thaliana* plants. **(A)** Phenotypes of plants infested with CiLV-C viruliferous *B. yothersi* mites, from 0 to 12 days after infestation (dai). Red dots in the top panel indicate symptomatic leaves. From 0 to 6 dai, no symptoms are observed. Typical symptoms of CiLV-C infection in *A. thaliana* initially arise as chlorotic spots in green dark leaves (7–9 dai), evolving to green islands in yellow senescent ones (10–12 dai). Alternatively, larger areas of dead tissues frequently appear in infected plants (here, represented by the last leaf from the 10–12 dai group). No symptoms are observed in plants infested with nonviruliferous mites (mock). **(B)** Absolute quantification of CiLV-C *p29* and *RdRp* genes in eight time points after infestation with viruliferous *B. yothersi* mites. Different letters correspond to different copy numbers between the time points assessed (ANOVA and Student’s t-test, α < 0.05). **(C)** *p29/RdRp* ratio, calculated using the copy number from both molecules at each time point. Different letters correspond to different ratios between the time points assessed (ANOVA and Student’s t-test, α < 0.05). Three stages (i: 0–24 h after infestation, ii: 2–4 dai, iii: 6–10 dai) from CiLV-C infection are indicated.

CiLV-C RNA loads were quantified during a time-course experiment in which leaf samples from *A. thaliana* plants were collected at ¼, ½, 1, 2, 4, 6, 8, and 10 days after infestation (dai) with viruliferous *B. yothersi* mites. Up until 6 dai, no symptoms were observed (**Figure 1A**). Symptoms of CiLV-C infection began appearing at 7 dai in 100% of the infested plants that were kept until the two latest time points. Typical symptoms of CiLV-C infection in Arabidopsis initially arise as chlorotic spots easily distinguished in green dark leaves, evolving to green islands in yellow senescent ones (**Figure 1A**) that may contain small patches of dead cells, as detected with Trypan Blue staining (Arena et al., 2016). Alternatively, areas of dead tissues with up to 5 mm in diameter appear in the infected leaves (**Figure 1A**). In both cases, these leaves undergo an accelerated senescence process leading them to death after 10–12 dai. Such a pattern of symptom development upon CiLV-C infection was previously reported in *A. thaliana* plants (Arena et al., 2013) and conforms to, but in a shorter temporal scale, those observed in CiLV-C-infected citrus species (Bastianel et al., 2010). Although not all the chlorotic spots give rise to necrotic ones, regardless of the final phenotype of the lesions, infection and symptoms are always restricted to the leaves infested by viruliferous *Brevipalpus* mites, and they are detected neither in systemic leaves nor in plants infested by nonviruliferous mites (**Figure 1A**).

Absolute quantities of *p29* and *RdRp* containing-RNA molecules were determined using the TaqMan assays and suitable standard curves (**Figure 1B**). All samples were positive in the analyses of both targets, confirming CiLV-C infection. The levels of both targets were kept invariable during the first 24 h after the infestation (hai), but afterward, they increased continuously until the last time point [Tukey’s *honest significant difference* (HSD) test, *α* ≤ 0.05]. The highest difference between the evaluated sequential time points was obtained from 2 to 4 dai when RNA molecules containing *p29* and *RdRp* increased 66- and 53-fold, respectively (**Figure 1B**).

CiLV-C replication analysis was expanded to cover the RNA2 viral molecule by using intercalating dye-based RT-qPCR assays (**Supplementary Figure 1**). PCRs targeted sequences within ORFs *p15*, *p61*, *p24, MP, p7* (putative small ORF downstream of *p15*), and the intergenic region (IR) (Locali-Fabris et al., 2006). Additionally, specific primers for ORFs *p29* and *RdRp* were also included in this analysis to compare the data with those obtained in the TaqMan-based assays. Even though the absolute amounts of the molecules were not quantified in the intercalating dye-based RT-qPCR assays, they tended to follow the same pattern described by *p29* and *RdRp* in the TaqMan-based assays. Genes *p15*, *p61*, *p24*, and *MP* were mostly invariable within the first 24 h and continuously increased onwards (**Supplementary Figure 1**).

The ratio *p29*/*RdRp* at each time point was calculated as an indicator of the accumulation of viral subgenomic and genomic RNA (**Figure 1C**). The number of molecules containing the ORF *p29* was higher than those containing the ORF *RdRp* across the whole experiment (**Figures 1B, C**). Higher accumulation of *p29* was expected as the assay detects both genomic (also including the antigenomic) molecules and the *p29* sgRNA, while *RdRp* assay quantifies only the genomic RNA1 molecule. Within 2–4 dai, the ratio *p29*/*RdRp* reached the lowest level, whereas before and after this period it showed similar levels (**Figure 1C**). Despite the asynchronism of the viral replication process over the infected cells, a heuristic approach of the experimental data allowed us to subdivide the CiLV-C accumulation in *A. thaliana* into three main kinetic steps: i) 0–24 h after infestation (hai), ii) 2–4 dai, and iii) 6–10 dai (**Figure 1C**). Considering these steps and pathobiology features inherent to the citrus leprosis disease, time points for the evaluation of the plant response to the viral infection were further selected, *i*.*e*., 6 hai, minimum inoculation access period required by viruliferous mites to obtain 100% of infected plants (Arena et al., 2016); 2 dai, first significant increase in the viral genome accumulation; and 6 dai, the presymptomatic stage when plant transcriptional responses likely take place culminating in the disease phenotype. Moreover, time points 2 and 6 dai epitomize a condition in which the ratio *p29*/*RdRp* significantly differs from the precedent kinetic steps.

### CiLV-C Infection Triggers Significant Transcriptome Changes Proportionally to the Increase of Viral RNA Loads

**Figure 2:**
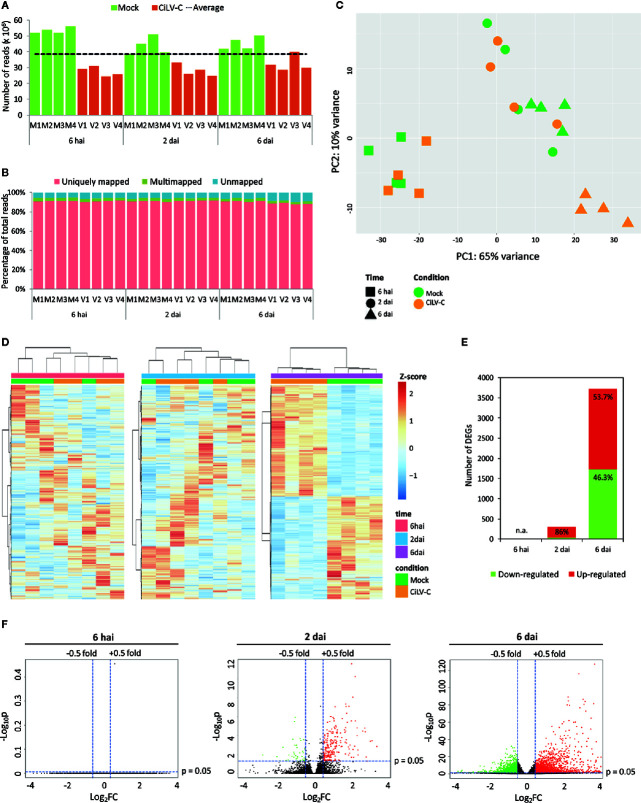
Overview of *Arabidopsis thaliana* transcriptome upon CiLV-C infection. **(A)** Number of paired-end reads generated for each library by Illumina HiSeq sequencing. M, mock-infected (plants infested with nonviruliferous mites); V, virus-infected (plants infested with CiLV-C viruliferous mites). The dashed line represents the average of paired-end reads from all 24 libraries. **(B)** Proportion of uniquely mapped, multimapped, and unmapped reads obtained for each library. Reads were mapped in the *A. thaliana* (TAIR 10) genome using *TopHat2*. M, mock-infected; V, virus-infected plants. **(C)** Principal component analysis of normalized count data from all samples. **(D)** Hierarchical clustering analysis of normalized count data z-scores exhibited by differentially expressed genes (DEGs) of each sample within each time point. **(E)** Numbers of up- and downregulated DEGs in CiLV-C infected plants in comparison to mock controls at each time point. DEGs were identified using *DESeq2* and defined by |log_2_FC| ≥ 0.5 and false discovery rate (FDR)-corrected *p*-value ≤ 0.05. **(F)** Volcano-plots of −log_10_p and log_2_FC exhibited by each gene in CiLV-C infected plants compared to mock controls at each time point. Up- and downregulated genes are presented in red and green, respectively. FC, fold-change; p, FDR-corrected *p*-value; hai, hours after infestation; dai, days after infestation.

The global transcriptomic response of *A. thaliana* plants along the course of CiLV-C infection was assessed by RNA-Seq. Plants infested with CiLV-C-viruliferous mites were compared with those infested with nonviruliferous ones (mock) at 6 hai, 2 dai, and 6 dai. Illumina sequencing generated roughly 924 million paired-end reads, with an average of 38.5 million per library and a higher number of reads from the mock treatment (**Supplementary Table 1**, **Figure 2A**). Overall, 93.5% of the reads aligned to the *A. thaliana* reference genome, with a 90.6% average of uniquely mapped reads (**Supplementary Table 1**, **Figure 2B**). The CiLV-C infected samples from 6 dai had the highest percentage of unmapped reads probably due to the higher virus titer and consequently a higher number of reads mapping to the virus genome (**Figure 2B**).

The main sources of variability within samples were assessed by principal component analysis (PCA) using the normalized count data (**Figure 2C**). The first component, which accounts for 64% of the variance, separated the samples by both variables: time after infestation and virus treatment, and they reflected the intensity of stimuli. Mock samples from different time points grouped separately, most likely due to the differential expression associated with longer mite feeding periods, as previously described (Arena et al., 2018). At 6 hai, where the lowest viral RNA loads were detected, all samples grouped regardless of the virus presence. A single group comprising both infected and mock samples was obtained for samples collected at 2 dai, where the viral RNA loads are slightly higher than at 6 hai. Differences between the expression profiles from infected and mock samples might be masked by the massive transcriptome changes in response to the mite action (Arena et al., 2018). At 6 dai, where the highest RNA virus loads were reached, infected and mock treatment formed two separated groups. The hierarchical clustering of samples within each time point confirmed the clusterization profile obtained by PCA (**Figure 2D**).

Differentially expressed genes (DEGs) in virus-infected plants compared with mock-inoculated ones were assessed within each time point using the negative binomial-based DESeq2 package and *False Discovery Rate* (FDR)-correction of *p*-values for multiple comparisons. Overall, 3,892 DEGs [*α* ≤ 0.05, |log2 fold change (FC)| ≥ 0.5] were detected (**Supplementary Table 2**). No gene was differentially expressed at 6 hai (**Figures 2E, F**), which agrees with the similar expression profiles displayed by mock and virus-infected plants (**Figures 2C, D**). The number of DEGs progressively raised along the course of the infection (**Figures 2E, F**). At 2 dai, 294 DEGs were detected, of which, the majority (253 DEGs, ≅ 86%) were upregulated (**Figure 2E**). The largest number of DEGs throughout the interaction was detected at 6 dai, when CiLV-C infection deregulated 3,717 genes, evenly distributed in 1,995 (≅ 53.7%) up- and 1,722 (≅ 46.3%) downregulated DEGs (**Figure 2E**). This corresponds to more than 11% of all 33,602 *A. thaliana* genes being differentially expressed in response to CiLV-C at this particular time point. The analysis performed here shows that CiLV-C infection triggers a significant reprogramming on infected plants likely mirroring the course of viral replication.

### CiLV-C Infection Induces Cell Growth and HR-Related Processes and Represses Both the Plant Primary Metabolism and the JA/ET-Mediated Responses

**Figure 3:**
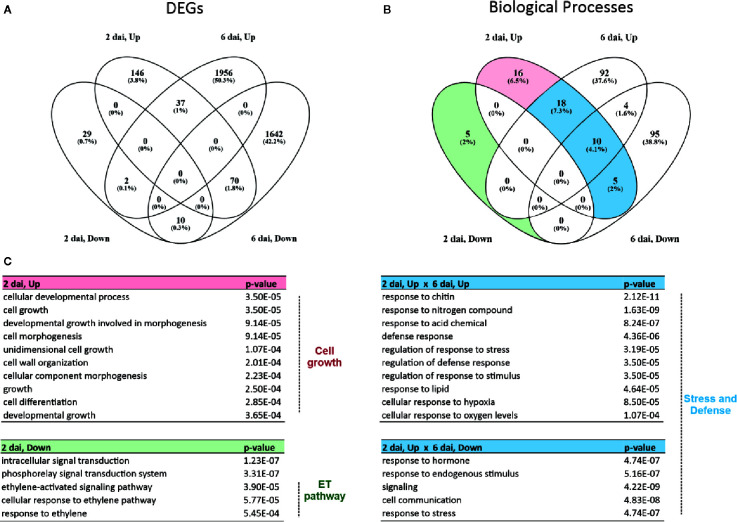
General transcriptomic changes and early responses of *Arabidopsis thaliana* plants affected by CiLV-C infection. **(A)** Venn diagram of up- and downregulated genes identified within the set of differentially expressed genes (DEGs) from each time point. DEGs were identified using *DESeq2* and defined by |log_2_FC| ≥ 0.5 and false discovery rate (FDR)-corrected *p*-value ≤ 0.05. The percentage value on each section of the diagram refers to the number of the corresponding DEGs relative to the total number of DEGs. **(B)** Venn diagram of overrepresented biological processes (BPs) from each set of up- and downregulated DEGs identified at each time point. Overrepresented BPs were identified based on a hypergeometric test with FDR-adjusted *p*-values ≤ 0.001. The percentage value on each section of the diagram refers to the number of the corresponding BPs relative to the total number of BPs. **(C)** Lists of overrepresented BPs exclusively modulated at 2 dai (transient early responses) or those common at 2 and 6 dai (stable early responses). The corresponding *p*-values obtained in the Gene Ontology (GO) enrichment analysis from 2 dai are included in the right column of each table. Up to ten BPs of each list are presented in each table. Complete lists of exclusive and common BPs are available in **Supplementary Table 3**. Due to the high number of BPs exclusively modulated at 6 dai, they were omitted from this figure and are presented in **Figure 4**. ET, ethylene.

Gene ontology (GO) enrichment analyses were performed with the independent sets of up- and downregulated DEGs from each time point to identify the most relevant biological processes (BPs) disturbed during CiLV-C infection (**Supplementary Table 3**). DEGs and BPs that were either shared or exclusive to the experimental sets are presented (**Figures 3A, B**). Using the Cytoscape app BinGO, the enriched BPs were visualized as networks where the color and size of each node identify the *p-*value and number of DEG of each ontology, respectively.

The GO enrichment analysis revealed 49 and 5 overrepresented BPs (hypergeometric test, *α* ≤ 0.001) in the sets of DEGs that were up- and downregulated at 2 dai, respectively. Even though most of the DEGs identified at 2 dai were exclusively induced at this time point (146 DEGs, **Figure 3A**), the majority of enriched BPs obtained from the set of upregulated genes at 2 dai overlapped between the induced sets of 2 and 6 dai (28 BPs, **Figure 3B**). This suggests that several processes triggered soon at 2 dai are still modulated a few days later, although with a different number of exclusive and shared DEGs, perhaps reflecting the occurrence of early and late responses of the same process. BPs enriched in both upregulated DEGs from 2 and 6 dai (stable early responses) included general terms of plant response to stimuli such as “defense response”, “regulation of response to stress”, and “regulation of response to stimulus” (**Supplementary Table 3**, **Figure 3C**). Likewise, enriched BPs that were upregulated at 2 dai and downregulated at 6 dai mainly referred to broad ontologies such as “response to hormone”, “response to endogenous stimulus”, and “signaling” (**Supplementary Table 3**, **Figure 3C**). On the other hand, 16 and 5 BPs were exclusive to the upregulated and downregulated DEGs, respectively, at 2 dai (transient early responses) (**Figure 3B**). The BPs uniquely induced at 2 dai were predominantly related to the cellular growth, *e*.*g*. “cell growth”, “cellular developmental process”, “cell wall organization”, “cell morphogenesis”, and “cell differentiation” (**Supplementary Table 3**, **Figure 3C**). Of the five BPs only repressed at 2 dai, three were associated with the ethylene pathway: “ethylene-activated signaling pathway”, “cellular response to ethylene stimulus”, and “response to ethylene” (**Supplementary Table 3**, **Figure 3C**).

Most of the detected BPs were overrepresented in those data sets with a higher number of genes, *i*.*e*. the ones modulated at 6 dai (**Figure 3A**). GO enrichment analysis disclosed 124 and 114 enriched BPs (hypergeometric test, *α* ≤ 0.001) in the groups of DEGs that were up- and downregulated, respectively, at 6 dai (**Supplementary Table 3**). Besides a few broad-term processes common to the DEGs induced at 2 dai, the vast majority of the BPs enriched at 6 dai were exclusively detected at that time point (late responses) (**Figure 3B**). Within these categories, only four were shared between the up- and downregulated-clusters corresponding to 6 dai, revealing that induced and repressed genes at that time point are mostly involved in different processes, and distinct pathways are differentially modulated in the presymptomatic stage (**Figure 3B**).

**Figure 4:**
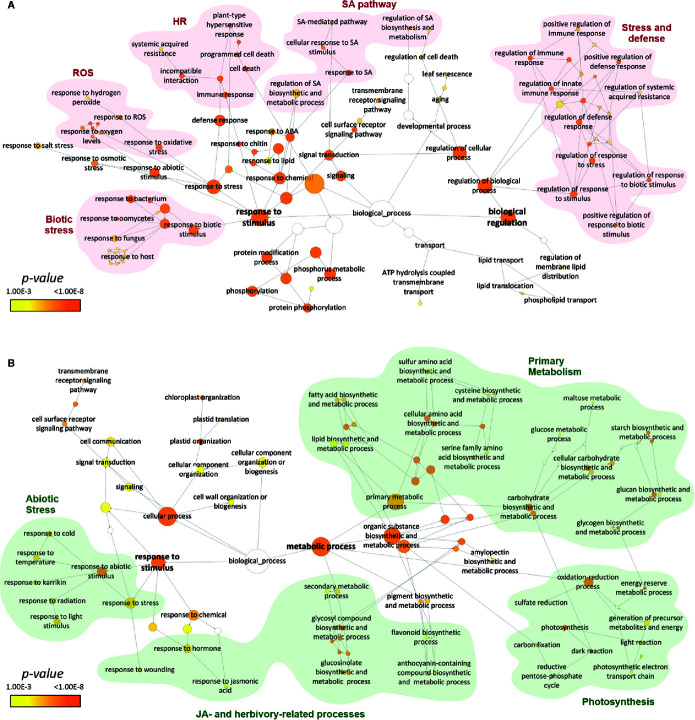
Responses of *Arabidopsis thaliana* plants affected by CiLV-C infection at 6 dai. Induced and repressed responses are represented as networks of enriched BPs from upregulated **(A)** and downregulated **(B)** DEGs generated using the app BinGO in Cytoscape. The size of the nodes correlates with the number of DEGs, and their color reveals *p*-values of the enriched categories. Names of some BPs were simplified for clarity; full names are displayed in **Supplementary Table 3**. HR, hypersensitive response; ROS, reactive oxygen species; SA, salicylic acid; JA, jasmonic acid; ABA, abscisic acid.

The cluster of upregulated DEGs at 6 dai was enriched in 92 exclusive categories (**Figure 3B**) and revealed a massive modulation of the plant immune system (**Figure 4A**, **Supplementary Table 3**). BP categories were mainly clustered in two groups comprising “response to stimulus” and “biological regulation” (**Figure 4A**). Processes associated with response to stress and defense preponderantly represented both groups. Particularly, the group centralized in “response to stimulus” was branched in stress-related nodes that included “response to biotic stimulus” (linked to the subcategories of response to bacteria, fungus, oomycetes, and host defenses), “response to abiotic stimulus” (represented by subcategories of response to osmotic stress and oxygen levels), and “response to oxidative stress” (specified from “response to ROS” to “response to hydrogen peroxide”) (**Figure 4A**). A defense-related branch from response to stimulus group displayed general ontologies (*e*.*g*. “immune response” and “defense response”), and it was typified by HR-related BPs such as “plant-type HR”, “defense response, incompatible interaction”, “systemic acquired resistance”, and “programmed cell death” (**Figure 4A**). BP group centralized in biological regulation was branched to a major subgroup comprising the ontologies related to the regulation of both responses to stress and defense (**Figure 4A**). Categories from that subgroup included general terms such as “regulation of defense response” and “regulation of response to stress” and others more specific, *e*.*g*. “positive regulation of response to biotic stimulus” and “regulation of systemic acquired resistance”. Another branch from the biological regulation group (“regulation of cellular processes”) displayed cell death- and SA-related responses including “regulation of cell death” and “regulation of SA biosynthetic and metabolic process”, respectively (**Figure 4A**). A small cluster associated with the senescence process was also present in the upregulated network (“aging”, “plant organ senescence”, and “leaf senescence”) (**Figure 4A**). Finally, the major hormonal-mediated pathway enriched in the upregulated network was the SA pathway, represented by the categories “response to SA”, “cellular response to SA stimulus”, and “SA mediated pathway” (**Figure 4A**).

The cluster of downregulated DEGs at 6 dai was enriched in 95 unique categories (**Figure 3B**). Most of the GOs clustered in a major group of metabolic processes harboring mainly the primary metabolism (**Figure 4B**). That subgroup included BPs associated with the metabolism of: *i*) lipids, such as “lipid biosynthetic and metabolic process” and “fatty acid biosynthetic and metabolic process”; *ii*) amino acids, whose categories included “sulfur”, “cysteine”, and “serine” amino acid biosynthetic and metabolic process; and *iii*) carbohydrate, with numerous broad terms (*e*.*g*. “cellular carbohydrate biosynthetic and metabolic process”) and specific BPs associated with biosynthesis and metabolism of glucan, starch, glycogen, and maltose. Carbohydrate-related processes were connected to a cluster of photosynthesis-related categories such as “photosynthesis, light and dark reaction”, “carbon fixation”, and “generation of precursor metabolites and energy”. Secondary metabolism formed a small branch comprising BPs directed to the biosynthesis and metabolism of glucosinolates and anthocyanins (**Figure 4B**), metabolites typically induced by JA during plant–arthropod interactions. Following the metabolism group, the downregulated GO network gathered a set of BPs, which along with many general terms shared with the upregulated network, included the response to distinct abiotic stimuli (“light”, “radiation”, and “temperature”), “response to wounding”, and JA as the only enriched hormonal pathway within the downregulated processes. Another small group from the network was centralized in the “cellular component organization or biogenesis”, with ontologies related to chloroplast and cell wall organization/biogenesis (**Figure 4B**).

Overall, the GO enrichment analysis showed that early plant responses to CiLV-C infection involve a transient induction of cell growth-related processes, transient repression of ET-responsive genes, and a stable modulation of defense and stress-related responses that kept up with the infection. At the presymptomatic stage, infected plants trigger processes related to the SA-mediated pathway, response to ROS and HR, all of which are present during incompatible interactions. Conversely, at the same stage, infected plants downregulate processes involved in the primary metabolism, JA-mediated pathway, and synthesis of glucosinolates.

### Regulation of Coexpressed Genes by Specific Classes of Transcription Factors (TFs) Correlates With Modulation of Stress Defense Responses

**Figure 5:**
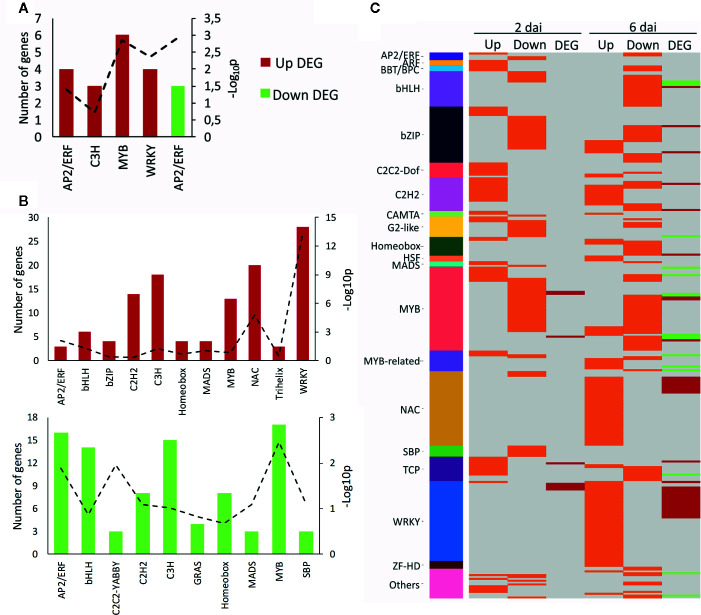
Enriched transcription factors (TFs) and TF targets during CiLV-C infection in *Arabidopsis thaliana* plants. **(A, B)** Number of up- and downregulated genes coding for TFs from each TF family identified within the set of DEGs at 2 dai **(A)** or 6 dai **(B)**. Families encompassing two or fewer TFs were omitted. Up- and downregulated DEGs are presented in red and green, respectively. Levels of enrichment (−Log_10_ *p*, with p: *p*-value) of each family (hypergeometric test, *α* ≤ 0.01) are presented by a dashed line with its corresponding values in the secondary axis. **(C)** TFs with enriched targets within each set of up- and downregulated DEG at 2 and 6 dai identified by the TF enrichment tool. TFs are grouped according to their families. Each line identifies one TF. Orange lines correspond to TFs with enriched targets within each set. Red and green lines represent up- and downregulated DEGs, respectively, encoding TFs at each time point. Gray lines indicate the absence of enriched targets for a given TF- and/or TF not differentially expressed. Families encompassing two or fewer TFs were grouped in “Others”. DEG, differentially expressed gene; dai, days after the infestation with viruliferous *Brevipalpus yothersi* mites.

To unravel the regulation of the transcriptional reprogramming upon viral infection, the classes of TFs associated with coexpressed DEGs were identified. First, we identified the up- and downregulated DEGs coding for TFs on each time point and their corresponding families (**Figures 5A, B**, **Supplementary Table 4**). The overrepresentation of specific families from each coexpressed set was assessed with a hypergeometric test (*α* ≤ 0.01). Within the set of upregulated DEGs at 2 dai, 29 TFs from 15 different families were identified. From that group, only two families were overrepresented: MYB (six genes, *p*-value = 1.39E-03) and WRKY (four genes, *p*-value = 4.46E-03) (**Figure 5A**), both typically involved in plant defense responses to stresses (Dubos et al., 2010; Phukan et al., 2016). From the downregulated genes at the same time point, only six TFs comprising three different families were detected, as expected due to the reduced number of DEGs with such expression patterns. The only overrepresented family was AP2/ERF (three genes, *p*-value = 1.16E-03) (**Figure 5A**), whose members are known to act as regulators of the ERF-branch of the JA/ET-mediated pathway (Pieterse et al., 2012). At 6 dai, 134 TFs from 22 families were upregulated. Only three of those families were overrepresented, of which the largest and most significant were WRKY (28 genes, *p*-value = 3.67E-14) and NAC (20 genes, *p*-value = 1.59E-05) (**Figure 5B**). Similar to WRKY, NAC TFs are also intimately associated with immune responses and specifically to increased resistance against pathogens, including the triggering of HR against viruses (Olsen et al., 2005; Nuruzzaman et al., 2013). Within the set of downregulated DEGs at 6 dai, 121 TFs evenly distributed in 31 families were identified. Similar to the upregulated set, only three classes of TFs were enriched, and the largest and most significant ones were MYB (17 genes, *p*-value = 3.50E-03) and AP2/ERF (16 genes, *p*-value = 1.31E-02) (**Figure 5B**).

In another approach, we searched for TFs with overrepresented targets within each set of DEGs by using the TF enrichment tool (Jin et al., 2017). Potential targets were identified based on cis-regulatory elements in the promoters of the test genes and regulatory interactions described in the literature (Jin et al., 2017) (**Figure 5C**, **Supplementary Table 5**). The largest families with potential targets within DEGs induced at 2 dai were the growth-related TCP (Manassero et al., 2013) and stress-related C2H2 (Kiełbowicz-Matuk, 2012), represented by 10 and 13 TFs, respectively. On the other hand, the families MYB and bZIP—with 29 and 18 TFs, respectively—were the ones that presented the highest numbers of TFs with potential targets in the set of downregulated genes at 2 dai (**Figure 5C**). Interestingly, the analysis of 6 dai sets revealed once again WRKY (43 TFs) and NAC (37 TFs) as the largest families with targets within upregulated DEGs (**Figure 5C**), supporting the involvement of both TF classes in controlling the induction of those genes. Within the downregulated genes at 6 dai, potential targets were mainly associated with TFs from bHLH (**Figure 5C**), which includes the regulators of the MYC-branch of the JA/ABA-mediated pathway (Pieterse et al., 2012) and MYB classes, represented by 33 and 17 genes, respectively. Neither members of WRKY and NAC families had targets enriched in the downregulated DEGs from 6 dai nor TFs from the bHLH family presented potential targets within the upregulated genes at the same time point (**Figure 5C**). This data stresses the specificity of induced and repressed responses during the presymptomatic stage.

Our analyses showed that the expression of upregulated genes is mainly driven by TF of the classes WRKY and NAC, followed by those of the TCP and C2H2, while downregulated genes are potentially controlled by TFs of the AP2/ERF, bHLH, and bZIP families. MYB TFs regulate both induced and repressed responses. Notably, WRKY is the only family with both modulated TFs and target genes exclusively within the groups of upregulated transcripts. Results support the modulation of stress responses upon CiLV-C infection, being consistent with the specific induction of cell growth and HR/SA-mediated defenses, and repression of both ET and ABA branches of the JA pathway.

### Genes Related to HR Are Induced at the Presymptomatic Stage

**Figure 6:**
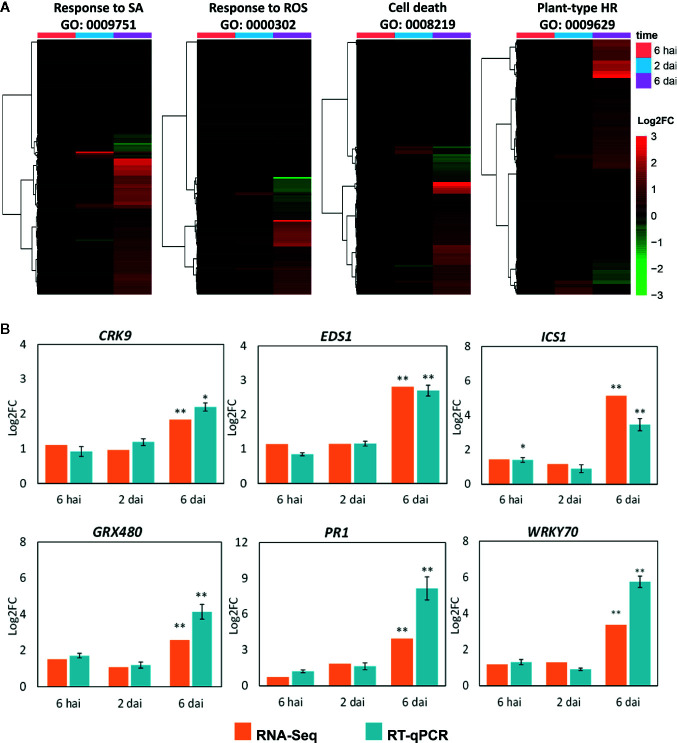
Hypersensitive response (HR)-related genes during plant infection by CiLV-C. **(A)** Hierarchical clustering analysis of the whole set of genes of the *Arabidopsis thaliana* genome assigned to the Biological Processes “Response to SA”, “Response to ROS”, “Cell death”, and “Plant-type HR”. **(B)** Expression profile of selected *Arabidopsis thaliana* genes in virus-infected plants, quantified by RNA-Seq and RT-qPCR. Data are presented as log_2_FC values in comparison with mock-infected plants (with log_2_FC set to zero). Statistically significant differences of virus-infected versus mock control at *p*-values ≤ 0.01 (**) and ≤ 0.05 (*) are indicated. Hai, hours after infestation; dai, days after infestation; GO, Gene Ontology term; SA, salicylic acid; ROS, reactive oxygen species; HR, hypersensitive response; FC, fold change.

Due to the overrepresentation of HR-related processes within the upregulated GO network and the HR-like phenotype induced by CiLV-C infection (Arena et al., 2016), the DEGs associated with either HR or the mechanisms underlying the development of such resistance response were thoroughly reviewed. Hierarchical clusters were generated with data from all *A. thaliana* genes included in the categories “response to SA”, “response to ROS”, “cell death”, and “plant-type HR” (**Figure 6A**). Within the sets of DEGs assigned to those categories (*α* ≤ 0.05, |log2FC| ≥ 0.5), 63, 34, 36, and 23 genes, respectively, were upregulated at 6 dai (**Supplementary Table 6**). The induction of several genes directly or indirectly related to HR at the presymptomatic stage provides additional evidence supporting the hypothesis that symptoms of CiLV-C infection result from an HR-like resistance.

To validate the RNA-Seq data and support the involvement of the SA pathway and HR in response to CiLV-C infection, the expression of selected DEGs was assessed by RT-qPCR (**Figure 6B**). Six SA- and HR-related genes upregulated at the presymptomatic stage were selected: the signaling component *EDS1* (*enhanced disease susceptibility 1*), the SA biosynthetic enzyme *ICS1* (*isochorismate synthase 1*), the regulator *GRX480* (*glutaredoxin 480*), the receptor-like kinase (RLK) *CRK9* (*cysteine-rich RLK 9*), the transcription factor *WRKY70* (*WRKY DNA-binding protein 70*), and the defense protein *PR1* (*pathogenesis-related protein 1*). Expression profiles of those genes were assessed in a new, independent, experiment with plants infested with nonviruliferous (mock) and CiLV-C viruliferous mites at 6 hai, 2 and 6 dai (**Figure 6B**). All the evaluated genes were induced at 6 dai, in line with the RNA-Seq data, supporting the results described in this work.

### Genes Involved in ER Stress and Unfolded Protein Response (UPR) Are Upregulated in CiLV-C-Infected Plants

Subcellular localization of CiLV-C proteins revealed that P15, P61, and P24 accumulate in association with the endoplasmic reticulum (ER) membranes, inducing disruption of the ER network (Leastro et al., 2018). To investigate whether the CiLV-C infection triggers ER stress, which could potentially induce HR-like response (Williams et al., 2014), we verified the expression levels of genes related to the ER stress and UPR activity. Genes assigned with the GO categories “endoplasmic reticulum unfolded protein response” and “response to endoplasmic reticulum stress” were reviewed. Nine and 17 genes included in each category, respectively, were differentially expressed, including the transcription factor *bZIP60* and the chaperones *ER luminal binding proteins* (BiP) and *calreticulins* (CRT). Without exception, all DEGs from both categories were upregulated at 6 dai (**Supplementary Table 7**). Even though the number of DEGs related to ER stress and UPR was not large enough to classify both GO terms as enriched ones, the upregulation of all DEGs at the presymptomatic stage suggests the involvement of these processes in the transcriptional changes that culminate in the development of the disease symptoms.

### Expression of CiLV-C P61 Protein Triggers a Hypersensitive-Like Response and Mimics Plant Responses to Viral Infection

The transcriptome analysis revealed that plant response to CiLV-C infection is spearheaded by the activation of the plant immune system, with marked induction of genes related to cell death, ROS production, SA pathway, and HR. We hypothesize that the induction of those defense processes leads to the HR-like response that characterizes the phenotype of the viral infection. Next, we investigated the phenomenon of the activation of the plant defenses under the viral perspective, by searching for CiLV-C components capable of triggering such responses in the infected plants. To unravel the role of CiLV-C-encoded proteins in triggering the plant responses identified in the transcriptome analysis, the six viral ORFs were cloned in expression vectors and individually expressed into *N. benthamiana* leaves by agroinfiltration. The putative elicitor activity of the viral protein was assessed by: i) visual inspection of leaf phenotypic characteristics, ii) histochemical detection of H_2_O_2_, the main ROS detected during plant–pathogen interactions, iii) evaluation of the expression profile of SA- and HR-related genes *pathogenesis-related 1* (PR1), PR2, *hairpin-induced 1* (HIN1) and *hypersensitive-related 203J* (HSR203J), and iv) quantification of the SA and JA hormonal contents.

**Figure 7:**
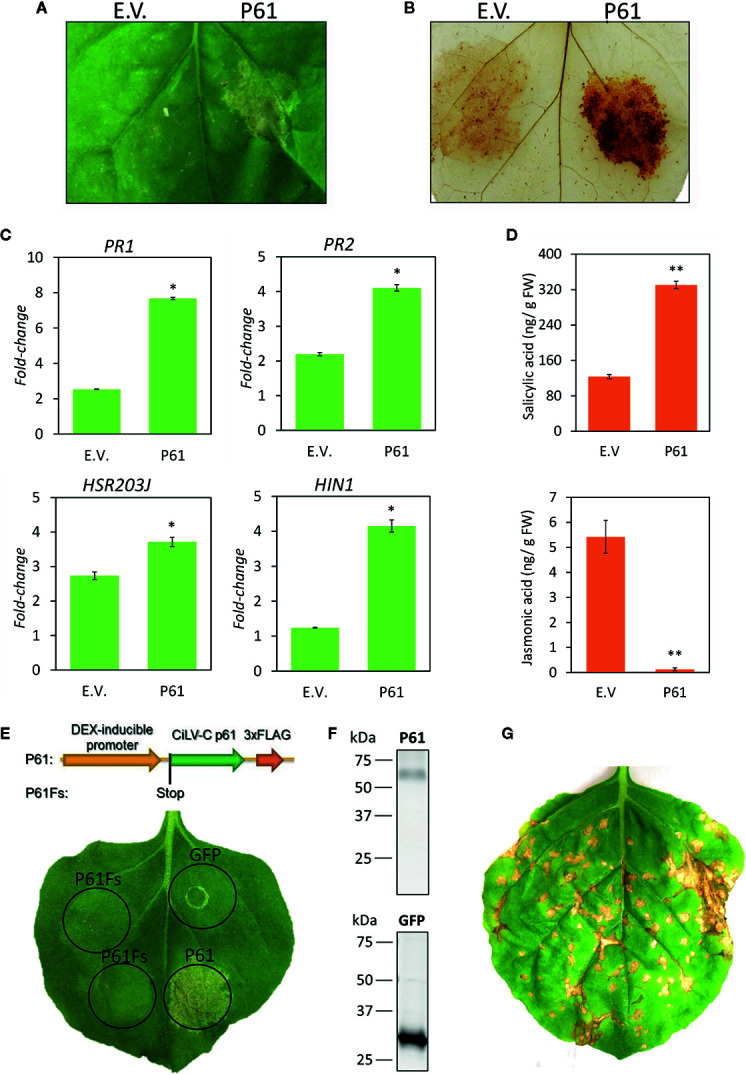
Elicitor activity of CiLV-C P61 protein. The viral protein was transiently expressed in *Nicotiana benthamiana* leaves using *Agrobacterium*-mediated infiltration. HR-like phenotype **(A)**, ROS production **(B)**, expression of SA- and HR-related genes **(C)**, and levels of jasmonic acid and salicylic acid **(D)** were assessed at 3 days after the infiltration. E.V., empty vector. Statistically significant differences of plants infiltrated with p61 *versus* empty vector (E.V.) at *p*-values ≤ 0.01 (**) and ≤ 0.05 (*) are indicated. To verify the elicitor activity of the *p61* RNA *versus* P61 protein, a frameshift mutant (P61Fs) was produced **(E)**. P61 or GFP 3xFLAG fusion proteins were detected in *N. benthamiana* leaf extracts by Western Blot probed with an antibody for the FLAG tag conjugated with HRP (anti-DYKDDDDK-HRP); expected sizes were 30 kDa for GFP-3xFLAG and 63 kDa for p61-3xFLAG **(F)**. Symptoms of CiLV-C in *N. benthamiana*, obtained by mechanical inoculation, are presented to highlight the HR-like phenotype **(G)**.

While the other CiLV-C proteins did not produce any altered phenotype (**Supplementary Figure 2**), the *Agrobacterium*-mediated transient expression of P61 consistently induced cell death on the infiltrated areas at 3 days after infiltration, which contrasted with the healthy phenotype observed in leaves infiltrated with both the *A. tumefaciens* carrying the empty vector and the infiltration buffer (**Figure 7A**). Histochemical analysis of the P61 infiltrated leaves revealed the production and accumulation of large amounts of H_2_O_2_ (**Figure 7B**). RT-qPCR assays showed that, although plants reacted to the infection by *A. tumefaciens* containing the empty vector, the presence of P61 clearly upregulated the expression of all the evaluated plant genes (**Figure 7C**). LC-MS/MS analyses revealed that SA levels were almost threefold higher on plants expressing this viral protein relative to those with the negative control, while JA levels were more than 42-fold lower (**Figure 7D**). Altogether, the results showed that the ectopic expression of the P61 protein triggers the same HR-related processes reported during plant infection with CiLV-C *i.e.* cell death, ROS production, SA pathway, and induction of HR-related genes. Mimicry of responses typically observed during CiLV-C-plant interaction indicated P61 as a viral effector that elicits plant defenses that likely culminate in the HR-like phenotype characteristic of the viral infection (**Figures 1A** and **7G**)

To verify whether the HR-like is triggered by either the P61 protein or its RNA sequence, a frameshift mutant preventing the production of the protein was produced (**Figure 7E**). With the insertion of two nucleotides (T and A) downstream the start codon of the *p61* ORF, the construction resulted in a modification of the ORF generating an *amber* stop codon immediately after the first codon. The clones carrying the genes for the *p61* frameshift mutant, the *p61* wild type, and that encoding GFP were agroinfiltrated in different spots throughout the same *N. benthamiana* leaf (**Figure 7E**). Cell death was observed in areas infiltrated with the *p61* wild type 3 days after infiltration. HR was observed in neither the areas infiltrated with *gfp* nor the *p61* frameshift mutant. Since the frameshift mutation did not affect the synthesis of the *p61* RNA, the result demonstrates that the HR-like phenotype is triggered only in the presence of the P61 protein.

## Discussion

In this work, we have dissected the interaction between plants and the kitavirid CiLV-C, a virus atypically unable to accomplish systemic infection in any of its known plant hosts, at the molecular level. The accumulation of CiLV-C genomic and subgenomic RNA molecules was coordinately studied with the transcriptome profile of infected plants to reveal the major mechanisms underlying the global plant response to the infection. Further, the transient expression of individual viral proteins allowed us to identify P61 as a putative viral effector causing the HR-like symptoms associated with the viral infection.

The analysis of the kinetics of viral RNA accumulation in *A. thaliana* plants infected by CiLV-C revealed the main features of an asynchronous process that we classified in three major steps (**Figures 1B, C**). The earliest stage occurs from the viral inoculation by viruliferous mites until 24 hai, which is characterized by a low number of viral molecules, low replication rate, or more likely, replication restricted to a few cells (**Figures 1B, C**). Accordingly, during the first 6 h of this stage, the plant transcriptional response was undetectable, at least with the experimental approach used here (**Figures 2E, F**). From 2 to 4 dai, the lowest difference between subgenomic and genomic RNA takes place (**Figures 1B, C**), suggesting a high replication rate, and a moderate number of plant transcriptional responses to viral infection is detected (**Figures 2E, F**). The third stage that ranges from 6 to 10 dai is marked by an increased accumulation of both subgenomic and genomic RNAs, reaching the highest detection levels (**Figures 1B, C**). Over the first hours of this step, which precedes the appearance of symptoms in *A. thaliana* leaves, a massive reprogramming of the plant transcript profile is observed (**Figures 2E, F**).

Early transcriptome changes in response to the infection by CiLV-C involve the upregulation of cell growth-related processes (**Figure 3C**). Induction of genes related to cell growth might be related to the development of hyperplasia or hypertrophy, histological changes typically promoted by phytopathogens, including several plant viruses (Hull, 2009). Likewise, symptomatic areas of sweet orange leaves with citrus leprosis show a higher division activity and larger size of the parenchyma cells (Marques et al., 2007). Hyperplasia and hypertrophy are also detected in citrus plant tissues infected by *Brevipalpus*-transmitted dichorhaviruses (Marques et al., 2010), suggesting that upregulation of cell growth responses might be a common pattern during the infection by *Brevipalpus* transmitted viruses (BTVs) in plants.

CiLV-C infection downregulated the interconnected ET and JA pathways at 2 and 6 dai (**Figures 3C** and **4B**), respectively. Similarly, downregulation of JA-responsive genes involved in the biosynthesis of glucosinolates, compounds acting against herbivores (Jander, 2014), was also detected at the last time point (**Figure 4B**). The concomitance of the induction of SA and reduction of JA during the plant infection by CiLV-C is likely a consequence of the SA-JA antagonism (Arena et al., 2016). The repression of antiherbivory defenses upon virus infection is used as a viral strategy to increase vector fitness or attraction and encourage virus transmission (Mauck et al., 2018; Donnelly et al., 2019; Carr et al., 2020). For instance, the polerovirus potato leafroll virus attenuates the induction of JA and ET by aphids, affecting vector fecundity and settling (Patton et al., 2020). By exploiting the natural SA–JA antagonism, the tospovirus tomato spotted wilt virus triggers the SA- to reduce JA-mediated defenses, rendering a more attractive host to the thrips vector *Frankliniella occidentalis* (Abe et al., 2012). Likewise, the induction of SA and reduction of JA pathways and related defenses upon CiLV-C infection might account for an improvement in *Brevipalpus* vector performance. In agreement with this hypothesis, CiLV-C infected *A. thaliana* leaves are preferred for mite colonization and oviposition (Arena et al., 2016), and experimental evidence suggests that mite density in CiLV-C infected sweet orange trees is higher than in the healthy ones (Andrade et al., 2013). Furthermore, the oviposition of *B. yothersi* mites in *A. thaliana* mutant plants compromised in SA signaling is lower (Arena et al., 2018), pointing out the role of SA response on the improvement of the mite performance. On this basis, we speculate that CiLV-C/*Brevipalpus* interaction is a mutualistic relationship, in which the better performance of mites on CiLV-C plants, showing a boosted SA response and likely a suppressed antiherbivore defense, improves the transmission rate of the virus.

Along with the reduction of the JA pathway and glucosinolate production, the presymptomatic stage during the CiLV-C infection was marked by a repression of the primary metabolism (**Figure 4B**). Detected downregulated processes in *A. thaliana* infected by CiLV-C comprised the metabolism of lipids, amino acids, and carbohydrates, including photosynthesis (**Figure 4B**). Inhibition of photosynthesis is a general rule across plant–virus interactions and is usually associated with changes in the chloroplasts and the development of chlorosis and necrosis (Goulart et al., 2019), some of the most common viral symptoms and a key feature in cilevirus infections. Chloroplasts are prime targets for plant viruses to help fulfill essential stages of viral infections such as replication and movement, undergoing massive structural and functional disturbance (Zhao et al., 2016; Bhattacharyya and Chakraborty, 2018). Conversely, chloroplasts play active roles in defense against viruses because they are the sites for the biosynthesis of SA and HR-related ROS, which might compromise photosynthesis (Zhao et al., 2016; Bhattacharyya and Chakraborty, 2018). Whatever the cause, plant viruses commonly damage chloroplasts, leading to reduced photosynthetic activity and development of chlorotic or even necrotic symptoms as a result of cell death (Zhao et al., 2016; Bhattacharyya and Chakraborty, 2018; Goulart et al., 2019). In this sense, the infection by CiLV-C seems to follow the commonly observed trend.

Even though the profile of some plant transcripts changed in the early stage of the CiLV-C infection, strikingly, the majority of the DEGs were identified at 6 dai (**Figure 3A**). As expected, the outcome of several biological processes identified at the presymptomatic stage might contribute to the development of the disease symptoms. Plant response at this phase of the viral infection is typified by the upregulation of the plant immune system (**Figure 4A**). A large number of upregulated genes are related to the SA-mediated pathway, response to ROS, cell death, and HR (**Figures 4A** and **6A**). In agreement with molecular responses detected in this work, histochemical analyses of tissues affected by CiLV-C revealed the accumulation of ROS and the presence of dead cells (Arena et al., 2016). In all its plant hosts, CiLV-C is restricted to cells around the vector inoculation sites where symptoms of viral infection arise (Freitas-Astúa et al., 2018; **Figures 1A** and **7G**). Phenotypically, these symptoms resemble the outcome of an HR, a cell death resistance process accompanied by pathogen restriction at the inoculation site during an incompatible interaction. Transcriptome changes associated with the induction of HR-like phenotype support the hypothesis that the lesions caused by citrus leprosis may be a consequence of an incompatible rather than a compatible interaction (Arena et al., 2016). Commonly, the development of an HR resistance is associated with the recognition of the viral protein by corresponding plant resistance (R) proteins in a host-specific manner (Garcia-Ruiz, 2019). It is noteworthy, however, that HR-like associated phenotypes (cell death and virus restriction) developed during CiLV-C infection occur over a large spectrum of CiLV-C hosts rather than in a specific plant species. Under these circumstances, alternative mechanisms leading to an HR-like phenotype cannot be ruled out.

Despite the fact that ER stress and UPR were not within the enriched GO terms, we identified the upregulation of genes related to both processes at the presymptomatic stage (**Supplementary Table 7**). Upon stress conditions, the accumulation of unfolded/misfolded proteins in the ER triggers the UPR, a protective response that improves protein folding activity and removes proteins from the ER (Afrin et al., 2020). When the relief of the ER stress fails, the programmed cell death can be activated (Eichmann and Schäfer, 2012; Williams et al., 2014). Virus-infected plants can upregulate UPR-related genes, and some virus-encoded proteins targeting ER induce UPR (Zhang and Wang, 2016). For instance, the TuMV protein 6K2 can induce UPR (Zhang et al., 2015), probably through its physical interaction with and remodeling of the ER (Laliberté and Sanfaçon, 2010; Zhang and Wang, 2016). Furthermore, evidence suggests that SA induces UPR in plants (Poór et al., 2019), and virus-induced ER stress triggers ROS production and Ca^2+^ influx that alert host defense systems (Zhang and Wang, 2016). Ultimately, ER stress may culminate in HR cell death during plant–virus infections (Williams et al., 2014). This is the case, for example, of the potato virus X movement protein, which activates the transcription factor bZIP60 to initiate the UPR and elicit programmed cell death (Ye et al., 2013). Similarly, the induction of UPR-related genes during CiLV-C infection raises the possibility that ER stress might take part in the processes triggering the plant immune system and HR-like cell death. Further studies are underway aiming to assess the contribution of the ER–UPR pathways during CiLV-C infection.

Finally, we showed that the transient expression of the CiLV-C P61 protein reproduces processes observed during plant interaction with CiLV-C, *i*.*e*. necrotic lesions in *A. thaliana* and *N. benthamiana*, and increased expression of HR-related genes, modulation of SA and JA pathways, and ROS burst in *A. thaliana* (Arena et al., 2016) (**Figure 7**). Besides, we demonstrated that all the tested processes are triggered by the expression of P61, but not by its mRNA (**Figure 7**). Several viral proteins that mimic responses of virus infection have been identified. For instance, the polerovirus P0 protein triggers HR necrotic lesions in *N. glutinosa* (Wang et al., 2015), the CP from cucumber mosaic virus interacts with a chloroplast ferredoxin protein causing chlorotic symptoms in tobacco (Qiu et al., 2018), and the *β*C1 protein from the beta satellite of tomato yellow leaf curl China virus interacts with the *MYC2* transcription factor decreasing levels of JA-responsive genes and enhancing the performance of its vector (Li et al., 2014). Induction of HR-like phenotype and related defenses brings CiLV-C P61 to the epicenter of processes triggering typical HR lesions. P61 causes structural remodeling of the ER membranes (Leastro et al., 2018), suggesting that the cell death caused by P61 expression might result from an unmitigated ER stress. Since ER stress signaling and UPR can be controlled by a nonfully known mechanism involving SA (Poór et al., 2019; Pastor-Cantizano et al., 2020), the interaction of P61 with *A. thaliana* or *N. benthamiana* plants might represent an interesting model for plant UPR regulation studies. Furthermore, due to the increased SA and reduced JA levels upon P61 expression (**Figure 7D**), it is suggested that P61 might trigger the cross-talk between hormonal pathways and modify the CiLV-C vector performance. New experimental approaches have been scheduled to verify the relevancy of these findings.

**Figure 8:**
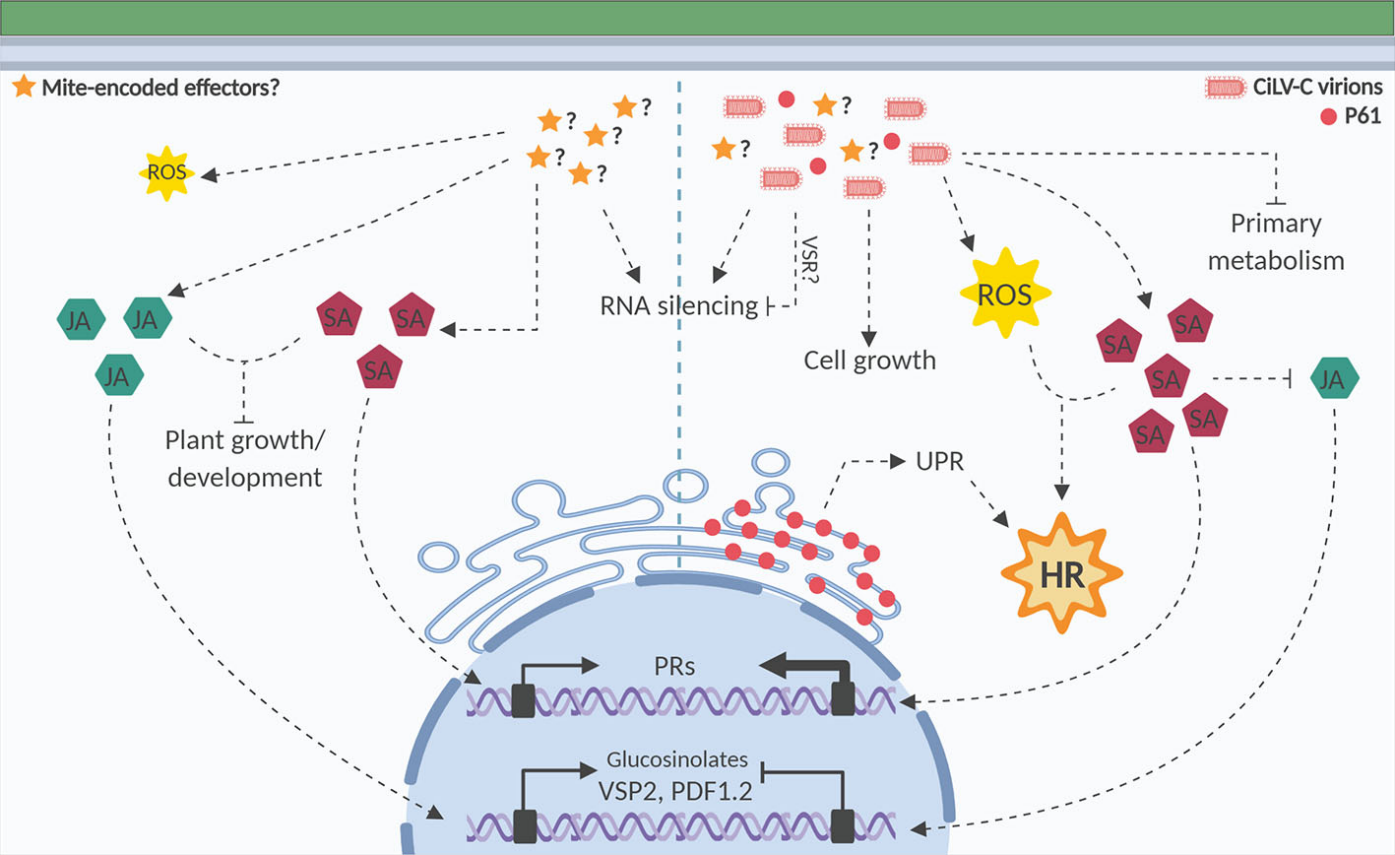
Model representing the interaction of *Arabidopsis thaliana* plants/*Brevipalpus* mites/CiLV-C. The current model integrates the experimental facts obtained in this work with those revealed from previous assays of interaction between *A. thaliana* plants and *B. yothersi* mites (Arena et al., 2016; Arena et al., 2018), and CiLV-C (Arena et al., 2016). Left and right halves of the diagram show hallmarks during the plant interaction with nonviruliferous and viruliferous mites, respectively. Left: During feeding, *Brevipalpus* mites use their stylets to pierce mesophyll cells and inject saliva that might contain mite-encoded effectors. In response to the interaction with mites, salicylic acid (SA) is accumulated and induces SA-dependent genes (*e*.*g*.: *pathogenesis-related proteins*, PRs), which benefit mite performance. In parallel, jasmonic acid (JA) is increased, triggering the expression of JA-dependent genes such as the ones involved in the synthesis of glucosinolates and the markers from the JA pathway VSP2 (*vegetative storage protein 2*) and PDF1.2 (*plant defensin 1.2*). RNA silencing and a reactive oxygen species (ROS) burst restricted to the few cells affected by mite feeding are also triggered. Plant growth and developmental processes are repressed (Arena et al., 2018). Right: Upon plant interaction with viruliferous mites, CiLV-C reaches mesophyll cells with mite salivary flow and moves locally to cells surrounding inoculation sites. Virus presence intensifies ROS production and the SA-mediated response and activates several genes related to cell death and hypersensitive response (HR). Those responses are also triggered by the transient expression of P61, placing this protein in the epicenter of processes triggering typical HR lesions; P61 protein accumulates in the ER membrane, which might cause an ER stress that triggers the upregulation of genes related to unfolded protein response (UPR) and contribute to the induction of cell death. CiLV-C infection also triggers the induction of genes associated with cell growth, which might be involved in hyperplasia and hypertrophy processes. JA response is repressed in virus-infected plants, probably as a consequence of the antagonism exerted by the increased SA production. Viral infection also inhibits the plant primary metabolism, *e*.*g*. photosynthesis, likely contributing to the development of the chlorosis symptoms. A putative virus suppressor of RNA silencing (VSR) may target and inactivate the first antiviral defense line, leading to the upregulation of a second line that enhances the RNA silencing activity (Arena et al., 2016). Ultimately, as a result of the activation of several defense mechanisms, CiLV-C remains restricted to cells around the mite feeding sites, where chlorotic/necrotic lesions develop as a probable consequence of an HR-like resulting from an incompatible interaction.

Results obtained in this work have enlarged and strengthened the previously proposed model depicting the plant response to components of the citrus leprosis pathosystem *i*.*e*. CiLV-C, and *Brevipalpus* mites (Arena et al., 2016; **Figure 8**). Comprehensively, we have provided host transcriptome data and viral protein expression evidence supporting the current understanding that the symptoms of CiLV-C infection arise from an HR-like resistance. CiLV-C is likely unable to overcome the myriad of plant defenses activated upon plant/virus interaction, which might prevent viral spread, thus restraining the viral infection in patches of tissues around the mite’s feeding/inoculation foci, where programmed cell death is further triggered. Although offbeat within the universe of known plant-infecting viruses, local lesions and the lack of systemic movement are common across members of the family *Kitaviridae* (Quito-Avila et al., 2020). Likely derived from a common ancestor with nege and nege-like viruses (Ramos-González et al., 2020), lack of systemic infection of kitavirids in plants might be a consequence of their unfitness in plants, *i*.*e*. failure to circumvent the plant defenses, which indirectly suggests a relatively short coevolutionary history of the biosystem kitavirus–plant. While the specific involvement of the innate immunity, including the putative existence of an R gene, UPR mechanism, and gene silencing on the kitavirus infection has been pointed out, the role of abiotic factors, such as temperature, on the pathosystem cannot be ruled out. Plant rhabdoviruses of the genus *Dichorhavirus*, which are also transmitted by *Brevipalpus* mites and produce nonsystemic infection under natural conditions, show a striking ability to infect plants systemically when incubated above 28°C (Dietzgen et al., 2018). Alternatively, since the rewire of the plant hormone metabolism seems to be relevant for *Brevipalpus* mite performance on CiLV-C infected plants, and on the other hand, the resistance breaking may affect virus fitness *i*.*e*. transmission and survival (García-Arenal and Fraile, 2013), a valid question would be what is the actual contribution of HR-like resistance to the fitness of this pathosystem. Ongoing experiments to clarify these and other aspects of CiLV-C plant and vector interplays, *e*.*g*. whether cilevirus multiply in their mite vector, will help to shed light on the forces and boundaries shaping the kitavirus evolutionary history.

## Data Availability Statement

The RNA-Seq raw data are available at sequence read archive (SRA) with the ID PRJNA454529.

## Author Contributions

GA and PR-G conceptualized and wrote the original draft. GA and PR-G worked on the formal analysis. JF-A, BF, CC, and MM supervised the study. JF-A, BF, CC, and MM were responsible for funding acquisition. GA, PR-G, CC, and JF-A worked on the investigation and methodology. All authors contributed to the article and approved the submitted version.

## Funding

The authors are grateful to CAPES and FAPESP for scholarships and research grants associated with this work (CAPES PNPD20132154–33141010001P4–PNPD–IBSP, 88882.157041/2017-01; FAPESP 2014/50880-0, 2014/00366-8, 2016/23870-9, 2017/50222-0, and 2019/02137-0). This study was financed in part by the Coordenação de Aperfeiçoamento de Pessoal de Nível Superior—Brazil (CAPES) Finance Code 001.

## Conflict of Interest

The authors declare that the research was conducted in the absence of any commercial or financial relationships that could be construed as a potential conflict of interest.

## Supplementary Material

Supplementary Figure 1 Accumulation of CiLV-C genes through the course of viral infection in *Arabidopsis thaliana* plants. Normalized relative quantities (NRQs) of molecules were determined by RT-qPCR at eight time points after infestation with viruliferous *Brevipalpus yothersi* mites. Data are presented as log_10_NRQ values in comparison with the time point with the lowest quantity (with log_10_NRQ set to zero). Different letters correspond to different quantities between the time points assessed (ANOVA and Student’s t-test, *α* < 0.05). *RdRp*, RNA-dependent RNA polymerase; *MP*, movement protein; *IR*, intergenic region; h, hours after infestation; d, days after infestation.

Supplementary Figure 2 Phenotype resulted from the expression of CiLV-C proteins in *Nicotiana benthamiana* plants. The viral proteins were transiently expressed in *N. benthamiana* leaves using *Agrobacterium*-mediated infiltration. Left half (I) of each leaf was infiltrated with *A. tumefaciens* GV3101 containing the empty vector, while right half (II) was infiltrated with the bacteria containing the construct for the expression of the corresponding CiLV-C protein. A: *p61*, B: *RdRp* motif, C: *p24*, D: *p15*, E: *mp*, F: *p29*, and G: *methyltransferase* motif.
