# Supplementary figures and images for: Plant Immune System Activation Upon Citrus Leprosis Virus C Infection Is Mimicked by the Ectopic Expression of the P61 Viral Protein
Source: Front Plant Sci. 2020 Aug 7;11:1188. doi: 10.3389/fpls.2020.01188 (PMC7427430; doi:10.3389/fpls.2020.01188)

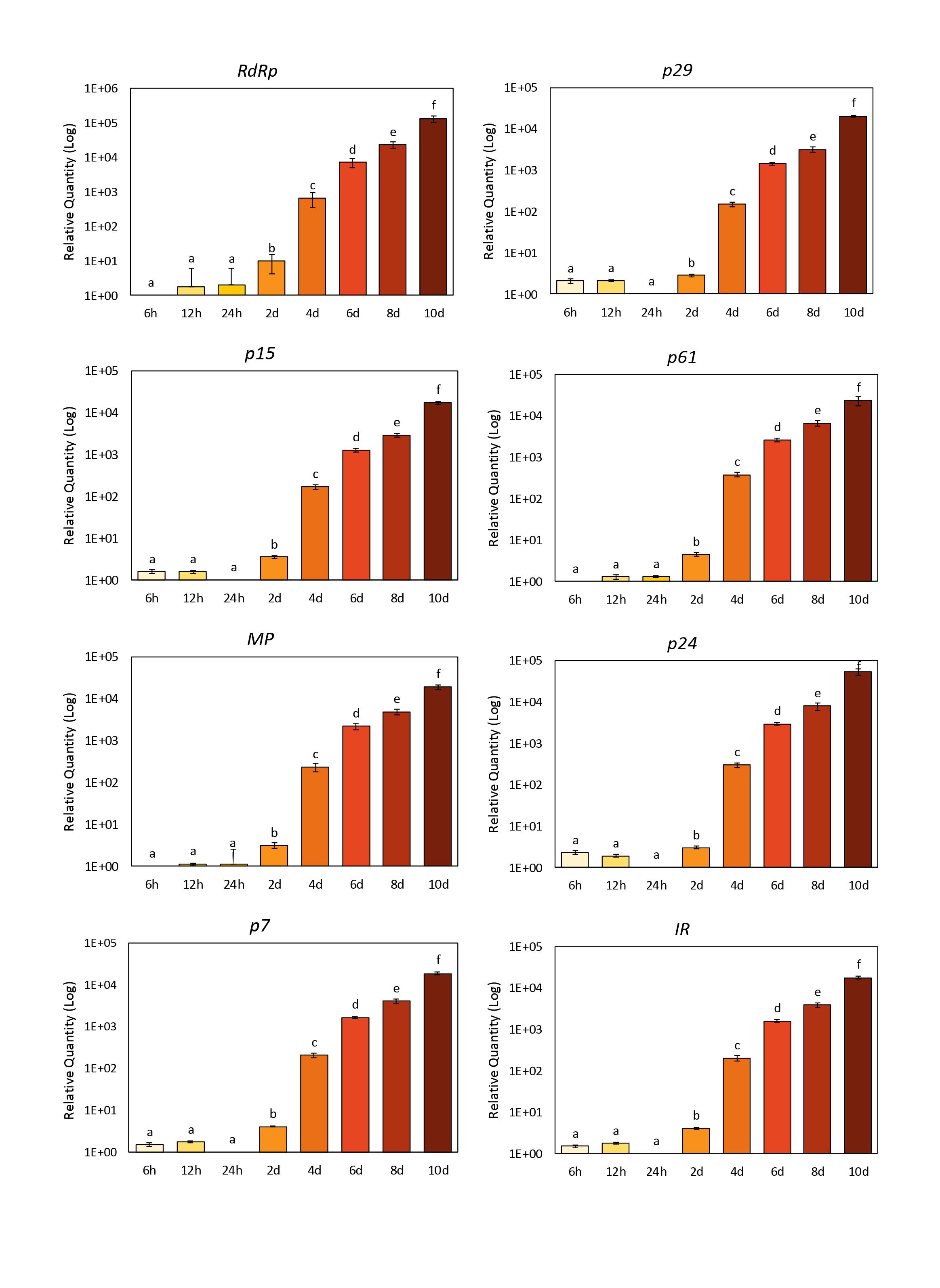

Supplement: Supplementary Figure 1 — Accumulation of CiLV-C genes through the course of viral infection in Arabidopsis thaliana plants. Normalized relative quantities (NRQs) of molecules were determined by RT-qPCR at eight time points after infestation with viruliferous Brevipalpus yothersi mites. Data are presented as log10NRQ values in comparison with the time point with the lowest quantity (with log10NRQ set to zero). Different letters correspond to different quantities between the time points assessed (ANOVA and Student’s t-test, α < 0.05). RdRp, RNA-dependent RNA polymerase; MP, movement protein; IR, intergenic region; h, hours after infestation; d, days after infestation. [file Image_1.jpeg]

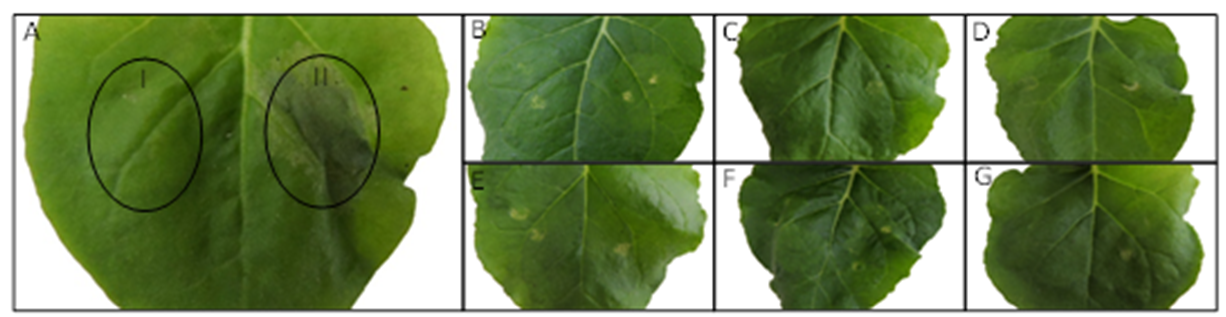

Supplement: Supplementary Figure 2 — Phenotype resulted from the expression of CiLV-C proteins in Nicotiana benthamiana plants. The viral proteins were transiently expressed in N. benthamiana leaves using Agrobacterium-mediated infiltration. Left half (I) of each leaf was infiltrated with A. tumefaciens GV3101 containing the empty vector, while right half (II) was infiltrated with the bacteria containing the construct for the expression of the corresponding CiLV-C protein. A: p61, B: RdRp motif, C: p24, D: p15, E: mp, F: p29, and G: methyltransferase motif. [file Image_2.tif]
