# Supplementary material for: Plant Immune System Activation Upon Citrus Leprosis Virus C Infection Is Mimicked by the Ectopic Expression of the P61 Viral Protein
Source: Front Plant Sci. 2020 Aug 7;11:1188. doi: 10.3389/fpls.2020.01188 (PMC7427430; doi:10.3389/fpls.2020.01188)
Supplement: Supplementary file 3 [file Table_1.docx]

**Supplementary Table 1.** Sequencing and alignment statistics for all *Arabidopsis thaliana* samples. hai: hours after infestation, dai: days after infestation.

| **Samples** | | **Total reads** | | **Mapped reads** | | **Mapped (%)** | | **Uniquely mapped (%)** | **Multimapped (%)** |
| --- | --- | --- | --- | --- | --- | --- | --- | --- | --- |
| 6 hai | Mock 1 | | 51,828,306 | | 48,872,195 | | 94.3 | 91.0 | 3.3 |
|  | Mock 2 | | 53,937,770 | | 50,967,558 | | 94.5 | 91.4 | 3.1 |
|  | Mock 3 | | 51,890,962 | | 49,001,322 | | 94.4 | 91.3 | 3.2 |
|  | Mock 4 | | 56,200,284 | | 53,208,550 | | 94.7 | 91.4 | 3.2 |
|  | CiLV-C 1 | | 29,114,872 | | 27,171,917 | | 93.3 | 90.3 | 3.0 |
|  | CiLV-C 2 | | 31,213,746 | | 29,289,486 | | 93.8 | 90.6 | 3.3 |
|  | CiLV-C 3 | | 24,555,054 | | 23,177,723 | | 94.4 | 91.5 | 2.9 |
|  | CiLV-C 4 | | 25,798,598 | | 24,367,754 | | 94.5 | 91.6 | 2.8 |
| 2 dai | Mock 1 | | 38,755,886 | | 36,221,667 | | 93.5 | 90.7 | 2.8 |
|  | Mock 2 | | 45,170,082 | | 42,526,976 | | 94.1 | 91.2 | 2.9 |
|  | Mock 3 | | 50,900,656 | | 48,065,714 | | 94.4 | 91.4 | 3.1 |
|  | Mock 4 | | 39,623,954 | | 36,967,715 | | 93.3 | 90.4 | 2.9 |
|  | CiLV-C 1 | | 33,187,552 | | 31,254,693 | | 94.2 | 91.4 | 2.7 |
|  | CiLV-C 2 | | 25,958,942 | | 24,366,588 | | 93.9 | 91.1 | 2.8 |
|  | CiLV-C 3 | | 28,663,956 | | 27,044,045 | | 94.3 | 91.6 | 2.7 |
|  | CiLV-C 4 | | 24,695,318 | | 23,322,503 | | 94.4 | 91.7 | 2.8 |
| 6 dai | Mock 1 | | 41,967,600 | | 39,203,750 | | 93.4 | 90.7 | 2.7 |
|  | Mock 2 | | 47,551,420 | | 44,768,874 | | 94.1 | 91.4 | 2.8 |
|  | Mock 3 | | 42,260,866 | | 39,386,679 | | 93.2 | 90.4 | 2.8 |
|  | Mock 4 | | 50,360,450 | | 47,480,937 | | 94.3 | 91.5 | 2.8 |
|  | CiLV-C 1 | | 31,752,434 | | 28,994,076 | | 91.3 | 88.5 | 2.8 |
|  | CiLV-C 2 | | 28,447,316 | | 26,062,757 | | 91.6 | 89.0 | 2.6 |
|  | CiLV-C 3 | | 39,814,158 | | 35,971,814 | | 90.3 | 87.7 | 2.7 |
|  | CiLV-C 4 | | 29,771,728 | | 27,019,809 | | 90.8 | 88.1 | 2.7 |
| Average | | 38,475,912.9 | | 36,029,795.9 | | 93.5 | | 90.7 | 2.9 |
