# Supplementary material for: Plant Immune System Activation Upon Citrus Leprosis Virus C Infection Is Mimicked by the Ectopic Expression of the P61 Viral Protein
Source: Front Plant Sci. 2020 Aug 7;11:1188. doi: 10.3389/fpls.2020.01188 (PMC7427430; doi:10.3389/fpls.2020.01188)
Supplement: Supplementary file 10 [file Table_8.docx]

**Supplementary Table 8**. Selected genes and corresponding primer pairs of *Arabidopsis thaliana* used for gene expression analyses by RT-qPCR.

| Gene | Name | Locus | Primer (5´-3´) | Reference |
| --- | --- | --- | --- | --- |
| *CRK9* | *Cysteine-rich RLK 9* | AT4G23170 | F: AGAACGTCACGGTGGGACAAAC | (Xu et al., 2015) |
|  |  |  | R: TACTGCAAACTTCCGGCGAGAG |  |
| *EDS1* | *Enhanced disease susceptibility 1* | AT3G48090 | F: GCTCAATGACCTTGGAGTGAGC | (Behmüller et al., 2016) |
|  |  |  | R: TCTTCCTCTAATGCAGCTTGAACG |  |
| *GRX480* | *Glutaredoxin 480* | AT1G28480 | F: GATTGATGAGGAGAGGGAAGATG | This work |
|  |  |  | R: CTACATAAACCGCCGGTAACT |  |
| *ICS1* | *Isochorismate synthase 1* | AT1G74710 | F: GAGACTTACGAAGGAAGATGATGAG | (Chen et al., 2009) |
|  |  |  | R: TGATCCCGACTGCAAATTCACTCTC |  |
| *PR1* | *Pathogenesis-related gene 1* | AT2G14610 | F: GTGCCAAAGTGAGGTGTAACAA | (Lindermayr et al., 2010) |
|  |  |  | R: CGTGTGTATGCATGATCACATC |  |
| *SAND* | *SAND family protein* | AT2G28390 | F: AACTCTATGCAGCATTTGATCCACT | (Czechowski et al., 2005) |
|  |  |  | R: TGATTGCATATCTTTATCGCCATC |  |
| *WRKY70* | *WRKY DNA-binding protein 70* | AT3G56400 | F: GGAAGAAGACAATCCTCATCGT | (Von Saint Paul et al., 2011) |
|  |  |  | R: CGTTTTCCCATTGACGTAACT |  |
