# Supplementary material for: Plant Immune System Activation Upon Citrus Leprosis Virus C Infection Is Mimicked by the Ectopic Expression of the P61 Viral Protein
Source: Front Plant Sci. 2020 Aug 7;11:1188. doi: 10.3389/fpls.2020.01188 (PMC7427430; doi:10.3389/fpls.2020.01188)
Supplement: Supplementary file 11 [file Table_9.docx]

**Supplementary Table 9**. List of primers for amplification, cloning, and modification of CiLV-C ORFs and *gfp* gene. The NcoI-XbaI restriction sites (for conventional cloning in pUC19 vector) or attB recombination sites (for Gateway-based cloning in pDONR207 vector) are underlined. Extra nucleotides introduced to produce a frameshift mutation on the p61 sequence (p61Fs) are shaded

| Genomic region | Primer | Sequence (5`-3`) |
| --- | --- | --- |
| *p29*^a^ | Forward | ATGCCATGGGTATCGTAACTTTCACTTTGAC |
|  | Reverse | GCTCTAGATTACTGCGCTGAGTCG |
| *mp*^a^ | Forward | ATGCCATGGCTCTTTCTACCAATAAC |
|  | Reverse | GCTCTAGATTATTCGCTTGTAGAAGTTGAG |
| *p24*^a^ | Forward | ATGCCATGGACGCTCAACTTCTAC |
|  | Reverse | GCTCTAGATTAACCAAAGTTGACGG |
| *p61*^a^ | Forward | ATGCCATGGCGCTATTTCAGCT |
|  | Reverse | GCTCTAGATTACAAATCAATGGTTTCAGC |
| *p15*^a^ | Forward | ATGCCATGGTGAACTGGTCTACGATTG |
|  | Reverse | GCTCTAGATTAAGTCTCAAGATTATGCAG |
| *RdRp*^a*^ | Forward | ATGCCATGGACTTAGATTTAGCTATTGATGA |
|  | Reverse | GCTCTAGATTAAAAGAGCAACCTAAACTCG |
| *met*^a*^ | Forward | ATGCCATGGCTCTTCGTAAGAAACCC |
|  | Reverse | GCTCTAGATTATGTCGCTTCCCACTTC |
| *p61*^b^ | Forward | GGGGACAAGTTTGTACAAAAAAGCAGGCTTCATGGCGCTATTTCAGCTT |
|  | Reverse | GGGGACCACTTTGTACAAGAAAGCTGGGTCCAAATCAATGGTTTCAGC |
| *gfp*^b^ | Forward | GGGGACAAGTTTGTACAAAAAAGCAGGCTTCATGGTGAGCAAGGGCGAGG |
|  | Reverse | GGGGACCACTTTGTACAAGAAAGCTGGGTCCTTGTACAGCTCGTCCATGCC |
| *p61fs*^b^ | Forward | GGGGACAAGTTTGTACAAAAAAGCAGGCTTCATGTAGCGCTATTTCAGCTT |

^a^Amplicons cloned in pUC19 intermediary vector and further transferred to pCambia 2300 binary vector. Each transcriptional unit comprised the 35s cauliflower mosaic virus promoter driving constitutive expression of the viral ORF, the Ω fragment from TMV as a translational enhancer, the convenient CiLV-C ORF, and the *Nopaline synthase* terminator.

^b^Amplicons cloned in pDONR207 donor vector and further transferred by recombination to a Gateway-compatible version of the pTA7001 destination vector. Expression clones comprised 3xFLAG C-terminal tagged protein under the control of a dexamethasone-inducible promoter.

*For the *RdRp* ORF, the motifs were amplified instead of the complete ORF.
